# Markov chain Monte Carlo with Gaussian processes for fast parameter estimation and uncertainty quantification in a 1D fluid‐dynamics model of the pulmonary circulation

**DOI:** 10.1002/cnm.3421

**Published:** 2020-12-22

**Authors:** L. Mihaela Paun, Dirk Husmeier

**Affiliations:** ^1^ School of Mathematics and Statistics University of Glasgow Glasgow UK

**Keywords:** classification, emulation, Gaussian processes, MCMC, pulmonary circulation, uncertainty quantification

## Abstract

The past few decades have witnessed an explosive synergy between physics and the life sciences. In particular, physical modelling in medicine and physiology is a topical research area. The present work focuses on parameter inference and uncertainty quantification in a 1D fluid‐dynamics model for quantitative physiology: the pulmonary blood circulation. The practical challenge is the estimation of the patient‐specific biophysical model parameters, which cannot be measured directly. In principle this can be achieved based on a comparison between measured and predicted data. However, predicting data requires solving a system of partial differential equations (PDEs), which usually have no closed‐form solution, and repeated numerical integrations as part of an adaptive estimation procedure are computationally expensive. In the present article, we demonstrate how fast parameter estimation combined with sound uncertainty quantification can be achieved by a combination of statistical emulation and Markov chain Monte Carlo (MCMC) sampling. We compare a range of state‐of‐the‐art MCMC algorithms and emulation strategies, and assess their performance in terms of their accuracy and computational efficiency. The long‐term goal is to develop a method for reliable disease prognostication in real time, and our work is an important step towards an automatic clinical decision support system.

## Graphical abstract

Uncertainty quantification in a complex physiological model of the pulmonary circulation can be made computationally feasible by combining state‐of‐the‐art numerical Bayesian methods with statistical emulation using Gaussian Processes. A comparative assessment in terms of computational efficiency of several novel Bayesian (MCMC) algorithms identifies the most efficient algorithm among several state‐of‐the‐art alternatives, which could potentially be used for clinical decision making in real time. A multivariate classifier is trained to learn ‐ via an iterative algorithm ‐ to automatically handle unknown constraints in parameter space, caused by the violation of the physical model assumptions.

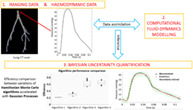

## 1 INTRODUCTION

Over the past few decades, cardiovascular modelling has seen an impressive advancement in terms of enabling better understanding of cardiovascular (patho)physiology, as well as assisting clinicians in the diagnosis, prognostication and treatment of cardiovascular diseases.^1^ Moreover, cardiovascular modelling can aid in surgical interventions, in the design and evaluation of medical devices, and even in the inference of unknown and immeasurable parameters from measured data.^1^ Before being used as a decision support system in the clinic, these models must first be adapted to patient‐specific conditions and an assessment of their credibility (uncertainty quantification, UQ) based on a comparison between model predictions and clinical data, must be performed. For example, studies in References 1 and 2 discuss the requirements that clinically applicable cardiovascular models must meet and the advances required to do so. Parameter inference and uncertainty quantification in biophysical models of physiological processes, and cardiovascular processes in particular is a challenging task to accomplish, as the physical models, typically expressed in terms of coupled non‐linear PDEs, with no closed‐form solution, are becoming more complex. This leads to a growing number of unknown biophysical parameters, which typically cannot be directly measured non‐invasively and hence have to be inferred from limited, potentially noisy clinical data, for which the acquisition process may be costly and require clinical personnel with expertise.

In the cardiovascular mathematical modelling community, most studies addressing parameter estimation focus on frequentist approaches with parameter optimisation, where the standard approach is to search for parameter configurations that lead to a good agreement between the clinical data and the predictions from the physical model by a minimisation of an objective function that quantifies the mismatch between predictions and data (typically the Euclidean distance).^3^, ^4^, ^5^, ^6^ However, an approach solely focused on parameter optimisation does not provide a direct mechanism for UQ. In contrast, sampling‐based Bayesian methods facilitate the uncertainty analysis via the exploration of the posterior distribution of the parameters given the available data, and can deal with the often multi‐modal and high‐dimensional posterior distributions of the parameters. However, sampling from the posterior distribution requires repeatedly calculating the likelihood by numerically integrating the PDEs, which renders the sampling process slow, and thus unattractive for clinical practitioners. In addition, finding efficient algorithms that return high effective sample sizes in a reasonable time frame is challenging, especially when there are strong posterior correlations among the parameters. Our study employs Bayesian methods and demonstrates how the issues outlined above can be overcome in practice, and how the parameter estimation and UQ can be made tractable.

The particular application of our study is a 1D fluid‐dynamics model of the pulmonary circulation,^6^ which predicts blood flow and pressure in the lungs by solving a system of non‐linear PDEs. The haemodynamic predictions, computed in an arterial network model constructed from micro‐computed tomography (CT) images from a control mouse, are compared to dynamic pressure data in the main pulmonary artery (MPA). The 1D model^6^ is characterised by two types of parameters: specifying the vessel network (i.e., vessel geometry or connectivity of the arteries), and the haemodynamics (i.e., pulmonary blood pressure and flow). The focus of this study is the inference of parameters describing the haemodynamic equations in a fixed vessel network obtained from one image segmentation. The parameters estimated are the vessel wall stiffness, which can only be measured ex‐vivo, and parameters specifying the PDE boundary conditions at each terminal vessel (resistance and capacitance of Windkessel models), that is, a total of four parameters.

Recently, a few cardiovascular modelling studies,^7^, ^8^, ^9^ which employ Bayesian (probabilistic) approaches, have emerged, but a lot of work still needs to be done in this field. In our previous work,^7^ we have shown how parameters of a system of PDEs describing the pulmonary circulation can be inferred using MCMC, the Delayed Rejection Adaptive Metropolis (DRAM) algorithm.^10^ This is referred to as the Bayesian analysis of computer code models, as the computer code defines a deterministic system that takes certain parameter values as input and outputs the data – for an overview of Bayesian calibration of computer models, see.^11^, ^12^, ^13^, ^14^ Similarly, Tran et al.^8^ employ an adaptive MCMC algorithm using differential evolution with self‐adaptive randomised subspace sampling (DREAM) for the coronary circulation. For a review on uncertainty and variability in computational and mathematical models of cardiac physiology, the reader is referred to.^15^ In addition,^16^ reviews the application of Bayesian methods to bioinformatics (e.g., protein informatics) and computational systems biology (e.g., quantitative network models). We also mention^17^, ^18^ for an application of MCMC to mathematical models for tumour growth. Although rare, in cardiovascular modelling, examples of studies employing MCMC to sample from multi‐modal distributions are^19^: with an application in soft tissue mechanics – constitutive modelling of tendon ligaments in mice, and^20^ with an application in haemodynamic models – modelling of pulmonary hypertension with a lumped parameter model on human data.

The MCMC algorithms can either be based on a random walk (RW, e.g., Metropolis‐Hastings^21^), or can introduce auxiliary variables for exploitation of gradient information from the posterior distribution (Hamiltonian Monte Carlo ‐ HMC^22^). All studies employing Bayesian methods in the cardiovascular modelling community use RW MCMC algorithms. However, the HMC algorithm has been shown to outperform the RW algorithms in terms of efficiency (e.g., for a 100‐dimensional multivariate Gaussian distribution as a target density, see Chap. 5 in References 23 or 24). Our study is the first to employ HMC‐type algorithms in cardio‐physiological modelling.

More generally, HMC has rarely been applied to non‐linear ODE or PDE models. Four noticeable exceptions are,^25^ where the authors applied HMC to infer the parameters of an ODE model of intracellular processes,^26^ where HMC is applied to a PDE‐based model of tumour growth, and,^27^ where an HMC extension (Riemann Manifold HMC) is applied to a PDE model of steady state heat conduction, or,^24^ where Hamiltonian Monte Carlo algorithms are applied to a set of ODEs describing dynamic causal models.

The principal disadvantage of applying HMC to inference in PDE models is the high computational cost associated with numerically integrating the PDEs a large number of times. HMC trajectories are simulated by following a set of deterministic Hamiltonian dynamics steps in parameter space. Throughout each trajectory, the PDEs are evaluated multiple times (for calculating the likelihood and its gradient) until a proposal is made, unlike the RW algorithms, which require one single PDE evaluation for a proposal to be made. For the calculation of the likelihood gradient, three approaches may be taken: finite differences,* forward sensitivities† (see the study in Reference 28 for a computation of sensitivity derivatives of Navier–Stokes equations in computational fluid‐dynamics), and the adjoint method‡.^24^, ^29^ For example, Sengupta et al.^24^ compare the performance in terms of computational speed and accuracy of the three methods on an ODE system, and the adjoint method was shown to be superior (specific details of these methods can also be found in Reference 29).

Regardless of the method chosen, calculating the likelihood gradients is very computationally intensive if performed repeatedly within the HMC algorithm. To address this shortcoming, two approaches have been proposed in the literature. One approach is a stochastic‐gradient HMC, introduced in Reference 30, and the main idea is to subsample the data, which introduces noise, and thus the full‐data gradients in the Hamiltonian equations are replaced by stochastic gradients, which can result in reduced exploration efficiency and accuracy.^31^ Another approach to speed up HMC is the use of computationally cheaper surrogate models for the expensive likelihood (or posterior distribution).^32^, ^33^ In Appendix A.1 we give a brief review of surrogate modelling.

HMC can be coupled with the surrogate model,^34^ and two main approaches can be taken to accomplish this: one which draws samples from the approximate posterior distribution defined by the surrogate model, and a second one which draws samples from the asymptotically exact posterior distribution. The first approach, taken by most studies on cardiac models,^35^, ^36^, ^37^, ^38^, ^39^, ^40^ and in the broader literature,^41^, ^42^, ^43^, ^44^, ^45^, ^46^, ^47^ samples from the posterior distribution induced by the surrogate model, which was constructed and refined prior to the sampling. While this leads to substantial gains in computational efficiency, it introduces a bias, that is, efficiency is gained at the expense of accuracy, as the inference relies heavily on the quality of the approximation model. Cotter et al.^48^ theoretically show that the bias could in principle be bounded, but no practical suggestions to do so are offered. Schiavazzi et al.^36^ verify that the bias introduced is small enough to be negligible in a surgery haemodynamics model for single ventricle palliation. This is accomplished by a forward UQ based on the parameter posterior samples obtained from the surrogate posterior distribution. The forward UQ allows to determine if the variability bounds on the model predictions are compatible with the expected measurement error. If the bias introduced by the emulator is too large, the model predictions may have unrealistically narrow variability bounds (bias‐variance trade‐off). This however relies on knowing the variance of the measurement error, which may not always be the case. To combat this issue, exact methods, in the sense of asymptotic convergence to the exact posterior distribution and can be used. One such method corrects for the bias by using the surrogate for the proposal only, for example, a study in Reference 32 couples HMC with a surrogate built using Gaussian Processes (GPs). Another study^33^ couples HMC with a surrogate created using random bases, that can scale better with high‐dimensional spaces than GPs. The latter study also introduces a method which refines the surrogate continually as MCMC proceeds (and thus reduces the number of training points required), while the asymptotic exactness is ensured. We also mention,^49^, ^50^, ^51^, ^52^ in which a similar approach is taken. For example, the approach in Reference 50 uses forward simulations from the PDEs for incrementally refining a local approximation of the (unnormalised) log posterior. However, while the authors prove the asymptotic consistency of their method, it depends on various heuristic parameters, which critically affect the computational efficiency and may be difficult to tune in practice. The Delayed Acceptance (DA)^53^, ^54^ scheme is another exact method, and its advantage is that it does not depend on any heuristically set terms, which motivates us to utilise it in our study. This method, employed in References 53, 54, 55, 56, 57, is a two‐stage acceptance procedure, with two separate acceptance/rejection decisions. The idea is that the first decision is a computationally fast pre‐filter step, which upon rejection of a proposed new parameter avoids having to carry out the computationally expensive second step.

In our work, we follow^32^, ^58^ and combine HMC§ with statistical emulation using GPs, a probabilistic inference method used to construct a surrogate model (known as the “emulator”) for the (unnormalised) log posterior distribution, and we implement this in a DA framework. Throughout the trajectory, the HMC algorithm is run at low computational costs on this surrogate function, and the Metropolis‐Hastings acceptance/rejection step uses the ratio of the true posterior distributions. This only requires the PDEs to be numerically integrated once throughout the HMC trajectory, and thus substantially reduces the computational complexity. The algorithm is exact in the sense of converging to the true posterior distribution, assuming no discretisation errors are introduced from the numerical integration of the PDEs (investigation of the discretisation errors is beyond the scope of this paper). Among cardiovascular mathematical modelling studies, we have found a single study in cardiac electrophysiology which uses a similar idea to ours: Dhamala et al.^61^ employ the Metropolis‐Hastings algorithm coupled with GPs and DA, and they take inspiration from a study by Lê et al.,^26^ who apply the HMC algorithm coupled with GPs to a tumour growth model. Both of these studies build a surrogate model for the log posterior distribution, and the latter also illustrates how an MCMC algorithm run on a surrogate model with no bias correction step can produce inaccurate inference results. However, these studies apply one single algorithm to the problem of interest, without any comparative evaluation of its performance.

Our first main contribution is to perform an extensive comparison of algorithms in terms of their efficiency and accuracy, and conclude with an algorithm recommendation. In addition, our inference procedure deals with unknown constraints in parameter space, caused by the violation of the physical model assumptions. In such problems, naively employing a GP‐MCMC method will yield inaccurate results, hence our second main contribution is the use of a multivariate classifier which automatically learns the infeasible parameter domain, and to our best knowledge, this is novel in the cardiovascular and mathematical biology research community. We therefore combine emulation with a series of state‐of‐the‐art sampling methods which converge to the true posterior distribution and are particularly adapted to complex models (i.e., models that are extensive to sample from). Since time is a critical factor for clinical decision making, we place a strong focus on computational efficiency. Thus, in our extensive benchmark study we look for the algorithm that provides the best trade‐off between accuracy and computational efficiency.

Our study presents the following methodological innovations. Firstly, we adapt the combination of GP emulation and HMC (henceforth referred to as the GPHMC algorithm), as proposed in Reference 32, to biophysical parameter estimation in a complex cardiovascular model describing the pulmonary circulation. We additionally include a GP classifier to deal with the a priori unknown regions in parameter space where the physical assumptions of the mathematical model are violated and the cardiovascular simulation software does not provide valid outputs. Secondly, we introduce the DA‐GPHMC algorithm, which couples the DA algorithm based on Sherlock's work^55^ with Rasmussen's GPHMC algorithm.^32^ Thirdly, we extend this framework to algorithms which advance HMC: No U‐turn sampler,^62^ Riemann Manifold HMC,^63^ and Lagrangian Dynamical Monte Carlo,^64^ and we automatically adapt the algorithms' tuning parameters with Bayesian optimisation, following an idea by Freitas in Reference 65. Fourthly, we perform a novel comparative evaluation of these algorithms in the application context of mathematical modelling of cardiovascular models.

The paper is structured as follows. Section 2 introduces the mathematical cardiovascular model and describes the data. Section 3 revises some background on statistical methodology, related to GPs and HMC. Section 4 discusses the new statistical methodology for the present work. Section 5 provides an overview of the setup of our simulation study. Section 6 presents our numerical results. Finally, Section 7 concludes with a discussion and an outlook on future work.

## 2 APPLICATION TO THE PULMONARY BLOOD CIRCULATION

This section describes the 1D fluid‐dynamics model utilised in this study, as well as the physiological data and synthetic data on which our inference procedure is carried out.

### 2.1 Fluid‐dynamics model

The particular focus of our work is a fluid‐dynamics model of the pulmonary blood circulation. This is part of a larger research project aiming to address the challenging task of non‐invasively diagnosing long‐term hypertension (high blood pressure) in the pulmonary system, that is, the blood vessel network connected to the right ventricle of the heart. Long‐term hypertension is a major risk factor for a variety of medical conditions, including coronary artery disease, stroke and heart failure. Non‐invasive measurement of the blood pressure in the systemic circuit, that is, the blood vessel network connected to the left ventricle of the heart, is straightforward; one just has to use a sphygmomanometer. However, sphygmomanometers cannot be applied to the pulmonary circuit. State‐of‐the‐art medical procedures are based on right‐heart catheterisation, which is an indispensable tool, providing important information about the function and structure of the measured physiological quantities. Catheterisation is typically performed once the symptoms of the disease have already appeared, and almost never used for screening and early diagnosis. Model‐based diagnostics used in the early stages of the disease could avoid a patient developing symptoms, and thus avoid the risks and possible side effects of right‐heart catheterisation (e.g., excessive bleeding because of puncture of the vein during catheter insertion, and partial collapse of the lung). The ultimate quest, therefore, is to combine magnetic resonance imaging (MRI) with mathematical modelling and statistical inference to be used as a disease diagnosis tool, likely with most impact in the early disease stages.

The present work is a first stepping stone in this direction. We use a 1D fluid‐dynamics model developed by the authors of.^6^, ^66^ Compared to 3D fluid‐dynamics models,^67^, ^68^ 1D models can predict nearly identical pressure and flow waves throughout the pulmonary network at a fraction of the computational cost, making them ideal as a real‐time clinical tool. Therefore, the 1D models are often used as computationally tractable approximations to 3D models.^38^ We use measured blood flow and pressure time series in the MPA to infer various biophysical parameters, including the vessel stiffness, which could provide important indicators for pulmonary hypertension. Subsequent studies will investigate this association more closely. The focus of the present study is the assessment of the accuracy and computational efficiency of the statistical inference and uncertainty quantification procedure.

**FIGURE 1:**
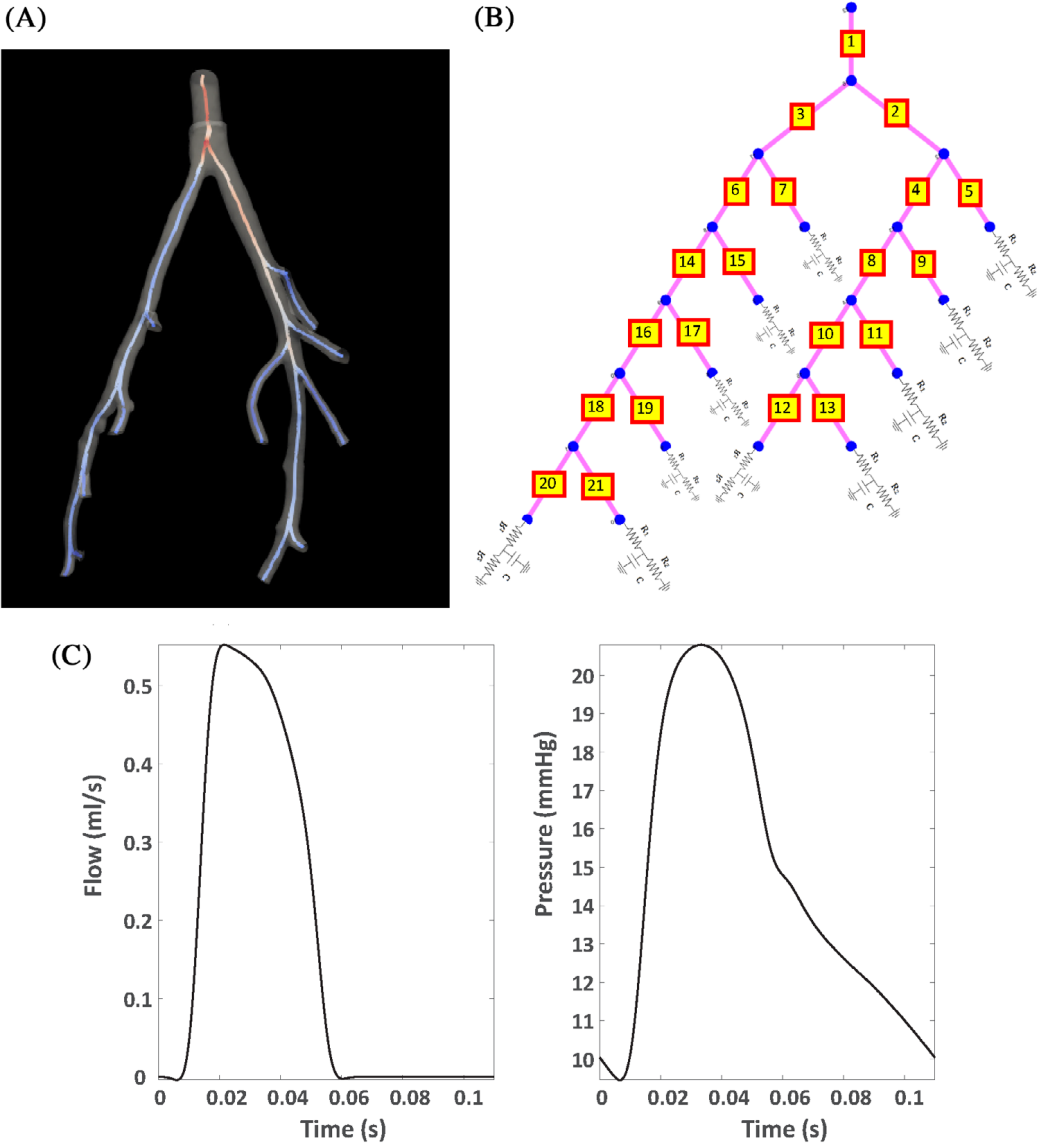
Panel (A): 3D smoothed network from a micro‐CT image of a healthy mouse lung. Panel (B): the connectivity graph of the same network. A three‐element Windkessel model with two resistors and a capacitor is attached at the end of each terminal vessel. Panel (C): Measured pulmonary blood flow (left) and pressure (right) in the main pulmonary artery of a healthy mouse. These data resulted from averaging of measurements across 20 heart beats (raw data are not available). The flow data are used as inlet boundary conditions in the PDEs (1)–(2), while the pressure data are used in the statistical inference procedure. Figures in panels (A) and (B) are taken from our study in 7

We apply the computational inference procedure discussed in the next section, with different MCMC variants, as reviewed in Section 3.2 to a 21‐vessel network model^6^, ^66^, which predicts 1D pulmonary arterial flow and pressure by solving a system of PDEs (see Figure 1 for a schematic of the arterial network of a healthy mouse lung). The model is derived from the incompressible axisymmetric Navier–Stokes equations for a Newtonian fluid, see Equation (1). The system of equations is closed with a constitutive equation describing the elasticity (i.e., stiffness) of the blood vessel walls, see Equation (2):

(1) $$\frac{\partial A}{\partial t}+\frac{\partial q}{\partial x}=0,\;\frac{\partial q}{\partial t}+\frac{\partial }{\partial x}\frac{q^{2}}{A}+\frac{A}{\rho }\frac{\partial p}{\partial x}=-\frac{2\mathrm{\pi \mu r}}{\delta }\frac{q}{A},$$

(2) $$p=p_{0}+\frac{4}{3}s\left(1-\sqrt{\frac{A_{0}}{A}}\right),$$

where *x*(cm) and *t*(s) are the longitudinal spatial and temporal coordinates, *p*(mmHg) is the blood pressure, *q*(ml/s) is the blood flow rate, *A*(cm^2^) is the cross‐sectional area, *A*_0_ is the unstressed vessel cross‐sectional area, *p*_0_(mmHg) is the transmural pressure, *s*(mmHg) is the arterial network stiffness, *ρ* = 1.055g/ml is the blood density, *μ* = 0.049 *g*/(cm s) is the viscosity and $\delta =\sqrt{\mathrm{\mu T}/2\pi \rho }\mathrm{cm}$ is the boundary‐layer thickness of the velocity profile. The boundary conditions are specified as follows. As inlet boundary conditions, an inlet flow at the MPA is prescribed, which is obtained from haemodynamic data. At the vessel junctions, conservation of flow ($q_{p}=q_{d_{1}}+q_{d_{2}}$) and continuity of pressure ($p_{p}=p_{d_{1}}=p_{d_{2}}$) are ensured; here p represents the parent vessel, and d_1_ and d_2_ represent the daughter vessels. As outflow boundary conditions, 3‐element Windkessel models (two resistors *R*_1_ and *R*_2_, and a capacitor, *C*) are used; they are attached at every terminal artery, and are of the form

(3) $$Z\left(\omega \right)=R_{1}+\frac{R_{2}}{1+\mathrm{i\omega}{\mathrm{CR}}_{2}}\Rightarrow q\left(L,t\right)=\frac{1}{T}\int_{0}^{T}p\left(L,t-\tau \right)Z\left(\tau \right)\mathrm{d\tau},$$

where *Z*(*ω*) is the impedance, *ω* is the angular frequency, *T* is the length of the cardiac cycle, *R*_1_, *R*_2_(mmHg s/ml) are the two resistances, and *C*(ml/mmHg) is the capacitance.

### 2.2 Model parameters

We follow^6^ and assume that the vessel stiffness *s*, is constant across the network – subsequent studies will relax this assumption. The three Windkessel elements (*R*_1_, *R*_2_, *C*) vary across the different terminal arteries. First, their nominal values are calculated for every terminal vessel *j* ($R_{01}^{j},R_{02}^{j},C_{0}^{j}$ in Equation (4)),^7^ then scaling factors *r*_1_, *r*_2_, *c* for these estimates are introduced, and they are kept constant across all terminal arteries:

(4) $$R_{1}^{j}=\left(1-0.5r_{1}\right)R_{01}^{j},\;R_{2}^{j}=\left(1-0.5r_{2}\right)R_{02}^{j}\;\text{and}\;C^{j}=\left(1-0.5c\right)C_{0}^{j}.$$

Thus, each outlet has different nominal values for the resistances and the capacitor, and all outlets are subsequently adjusted in the same manner.

The vector of parameters to be estimated is 4‐dimensional,

(5) $$\theta =\left(s,r_{1},r_{2},c\right).$$

These parameters lie within biologically plausible ranges (see Table 1), as established by the authors of^6^ based on the literature^69^ and the authors' experience running the mathematical solver. However, these ranges are univariate, and the parameters' behaviour in the joint space is not known prior to carrying out the analysis. Thus, we may need to deal with the fact that for certain parameter values and combinations, the underlying physical assumptions of the model are violated. Further details on this are given in Section 4.

**TABLE 1:** Univariate (prior) parameter ranges for the PDE parameters of the fluid‐dynamics model described in Section 2

Parameter	Range
*s*	[7 × 10^4^, 5 × 10^5^]
*r*_1_	[−0.5, 1.92]
*r*_2_	[−0.5, 1.0]
*c*	[−2.5, 1.5]

*Note:* Here *s* is the vessel stiffness parameter (expressed in g/cm/s^2^ units) and *r*_1_, *r*_2_, *c* are the Windkessel adjustment parameters (dimensionless).

### 2.3 Physiological data

The data for our study come from Qureshi et al.^6^ These are blood flow and pressure measurements in the arterial vessel network of a healthy mouse lung (see Figure 1). A summary of the experimental protocols used to extract the haemodynamic and image data can be found in References 6 and 66. For more detailed experimental protocols, the reader is referred to.^70^, ^71^ During heart contraction, blood is pumped into the arterial vessels. Waves of blood flow and pressure get propagated along the arterial circulation due to the pulsatile rhythm of the heart and the elasticity of the vessel wall. The blood flow is measured non‐invasively with ultrasound, and the pressure is measured invasively with right‐heart catheterisation.^70^ The raw data consisted of several time series of pulmonary blood pressure and flow measurements taken across 20 heart beats at one specific location in the MPA, which were averaged over the heart beats to obtain a single average time series covering one heart beat (the averaged blood flow and data can be seen in Figure 1(C)). The raw data for the 20 heart beats are not available. The flow measurements are used as inflow in the MPA (boundary condition for the PDEs). The parameter estimation is based on the pressure measurements.

### 2.4 Synthetic data

We simulate one data set from the mathematical model with parameter values consistent with the physiological data (i.e., the posterior median values), and to these data we add iid additive Gaussian noise, with the noise variance being the posterior median found based on the real data.

## 3 REVIEW OF RELEVANT STATISTICAL METHODOLOGY

This section provides a brief review of relevant methodological background. Our methodological innovation is presented in the next section, Section 4.

### 3.1 Gaussian processes

We use a regression GP model for statistical emulation of the unnormalised log posterior distribution^11^, ^72^, ^73^ (see Section 4), and a classification GP model to decide on the success of a PDE evaluation for particular parameter configurations. A GP model is a non‐parametric Bayesian technique, which we briefly review in Appendix A.2. More details on GPs can be found in Reference 74.

### 3.2 Gradient‐based MCMC algorithms

This section summarises the gradient‐based MCMC algorithms used in this study. We also refer to Table 2, which provides an overview of these algorithms and outlines the differences between them.

**TABLE 2:** Overview of the Hamiltonian and Lagrangian Monte Carlo algorithms used: Adaptive Hamiltonian Monte Carlo (AHMC), No U‐turn sampler (NUTS), Adaptive Riemann Manifold HMC (ARMHMC), Adaptive Lagrangian Dynamical Monte Carlo (ALDMC)

Algorithm	Auxiliary variables	Integration scheme	Parameter tuning method	2nd and 3rd order method	“mass matrix”
AHMC	“momentum”	Explicit leapfrog scheme	BO	No	Identity matrix
NUTS	“momentum”	Explicit leapfrog scheme	Stochastic optimisation for ɛ, *L* chosen recursively	No	Identity matrix
ARMHMC	“momentum”	Implicit generalised leapfrog scheme	BO	Yes	Negative Hessian matrix of log posterior
ALDMC	“velocity”	Explicit generalised leapfrog scheme	BO	Yes	Negative Hessian matrix of log posterior

*Note:* These algorithms augment the parameter space with an auxiliary variable. AHMC, NUTS and ARMHMC use “momentum” as an auxiliary variable, while ALDMC uses “velocity.” These algorithms simulate Hamiltonian or Lagrangian dynamics, that are numerically integrated. The integration scheme is based on the leapfrog method with a small step size ɛ and a number of steps *L* (called tuning parameters). The adaptive algorithms use Bayesian optimisation (BO) for parameter tuning, while NUTS has a different mechanism, see Section 3.2 for details. AHMC and NUTS use the first order derivative of the log posterior, while ARMHMC and ALDMC use also the second and third order derivatives. Thus, AHMC and NUTS set the “mass matrix” for the auxiliary variable (its covariance matrix) to the identity matrix, while for ARMHMC and ALDMC, the mass matrix is set to the negative Hessian matrix of the log posterior. The terms “momentum,” “velocity” and “mass matrix” are used for their mathematical equivalence to the corresponding terms in Hamiltonian mechanics.

***HMC*** . HMC^22^ is a powerful MCMC scheme which suppresses the random walk behaviour of Metropolis‐Hastings MCMC by introducing an auxiliary variable, the “momentum” variable, **r** ∈ ℝ^*d* × 1^, with density $p\left(r\right)=N\left(r|0,M\right)$, which guides the search towards high posterior density regions. HMC simulates Hamiltonian dynamics by using gradient information from the log target density.

Defining *H*: Hamiltonian, **r**: “momentum” vector, and ***θ***: “position” vector (representing our parameters that.

we want to infer), the Hamiltonian dynamic equations are:

(6) $$\frac{d\theta_{i}}{\mathrm{dt}}=\frac{\partial H}{\partial r_{i}},\;\frac{{\mathrm{dr}}_{i}}{\mathrm{dt}}=-\frac{\partial H}{\partial \theta_{i}}.$$

The Hamiltonian equations leave the Hamiltonian invariant, that is, *H*(θ^*^, r^*^) = *H*(***θ***, **r**),

(7) $$\frac{\mathrm{dH}}{\mathrm{dt}}=\sum_{i=1}^{d}\left(\frac{\partial H}{\partial r_{i}}\frac{{\mathrm{dr}}_{i}}{\mathrm{dt}}+\frac{\partial H}{\partial \theta_{i}}\frac{d\theta_{i}}{\mathrm{dt}}\right)=\sum_{i=1}^{d}\left(\frac{\partial H}{\partial r_{i}}\left(-\frac{\partial H}{\partial \theta_{i}}\right)+\frac{\partial H}{\partial \theta_{i}}\frac{\partial H}{\partial r_{i}}\right)=0.$$

The Hamiltonian dynamics (Equation (6)) are numerically integrated for a specified fictitious time. The leapfrog integrator^22^ is used, in which time is discretised using a small step size, ɛ > 0, and the trajectory is run for a number of leapfrog steps, *L*. The numerical integration induces an error, which implies that *H*(θ^*^, r^*^) no longer equals *H*(***θ***, **r**).

Further defining **M**: “mass matrix” for the “momentum” (its covariance matrix), *E*: “potential energy,” *K*: “kinetic energy,” we have:

(8) $$E\left(\theta \right)=-\left(\log p\left(\theta \right)+\log p\left(y|\theta \right)\right);\;K\left(r\right)=\frac{r^{T}M^{-1}r}{2};\;H\left(\theta ,r\right)=E\left(\theta \right)+K\left(r\right),$$

where log*p*(***θ***) and log*p*(**y**| ***θ***) are the log prior distribution of the parameters and the log data likelihood.

Note that the terms “mass matrix,” “momentum,” as well as “kinetic” and “potential energy” are used for their mathematical equivalence to the corresponding terms in Hamiltonian mechanics.

In statistical terms, the joint distribution is

(9) $$p\left(\theta ,r|y\right)=p\left(\theta |r,y\right)p\left(r\right)=p\left(\theta |y\right)N\left(r|0,M\right)\propto \exp\left(-E\left(\theta \right)\right)\exp\left(-K\left(r\right)\right)\propto \exp\left(-H\left(\theta ,r\right)\right).$$

Thus, if we denote the log posterior of the parameters we want to infer ***θ*** as log*p*(***θ***| **y**), then the negative log auxiliary joint distribution is:

(10) $$H\left(\theta ,r|y\right)=-\log p\left(\theta |y\right)+\frac{1}{2}\log\left({\left(2\pi \right)}^{d}\left|M\right|\right)+\frac{r^{T}M^{-1}r}{2}+\log Z,$$

where *Z* is a normalising constant and |**M**| is the determinant of the “mass matrix” **M**.

In HMC, at the end of each leapfrog trajectory (defined as a segment between 2 subsequent acceptance steps), a new point is proposed, and accepted with probability min(1, exp(−*H*(θ^*^, r^*^| **y**) + *H*(***θ***, **r**| **y**)). If the error from the numerical integration of the Hamiltonian dynamics in Equation (6) is small, then the acceptance probability will be high. If the new point is rejected, we keep the current point. The next trajectory is then simulated, and each trajectory starts with the resampling of the “momentum” variables from their marginal distribution $N\left(r|0,M\right)$, to allow properly being integrated out from the joint distribution, that is,

(11) $$p\left(\theta |y\right)=\int p\left(\theta |r,y\right)p\left(r\right)dr.$$

The HMC algorithm produces an ergodic, time reversible Markov chain, which satisfies detailed balance and whose stationary distribution is the marginal distribution *p*(***θ***| **y**).

In the classical HMC algorithm, **M** is kept fixed at the identity matrix, and the HMC tuning parameters, ɛ and *L*, are hand‐tuned in to get an acceptance rate >65% ^22^ and a large effective sample size.

Below we summarise extensions to the HMC algorithm, aimed at improving the algorithm's performance by automatic tuning of the HMC parameters or of the mass matrix **M**, to which the algorithm is known to be highly sensitive.

***RMHMC***. Riemann Manifold HMC (RMHMC)^63^ is an improved version of HMC, as it exploits the Riemannian geometry of the parameter space. It makes use of the curvature of the target distribution to set **M**, that is, **M** changes within every trajectory to adapt to the target density curvature (**M** → **M**(***θ***) in Equation (10)). We set **M** to be the observed Fisher Information matrix (the matrix of negative second order derivatives of the log likelihood), plus the negative Hessian matrix of the log prior. The joint distribution in Equation (9) is no longer factorisable. An implicit integrator is used (the Generalised Leapfrog algorithm), as proposals generated from the Leapfrog integrator no longer satisfy detailed balance in HMC: **M**(***θ***(*t*)) ≠ **M**(***θ***(*t* + ɛ)). The implicit integrator incurs high numerical costs. In RMHMC, ɛ and *L* are fixed.

***LDMC***. To overcome the increased computational costs in RMHMC associated with iteratively solving the equations of the implicit integrator, Lagrangian dynamics can be used instead of Hamiltonian dynamics. This leads to the Lagrangian Dynamical Monte Carlo algorithm ‐ LDMC.^64^ LDMC uses an explicit geometric integrator that replaces the “momentum” variable in RMHMC by “velocity” (used in analogy with classical mechanics), which improves the computational efficiency. However, the volume in phase space is no longer preserved, hence the Jacobian transformation is needed to adjust the acceptance probability to ensure detailed balance.^64^ In LDMC, **M** is adjusted to the curvature of the posterior distribution at every step throughout the trajectory, and ɛ and *L* are kept fixed.

***NUTS***. The No U‐turn sampler (NUTS), proposed in Reference 62, chooses *L* recursively by moving in parameter space until the HMC trajectory starts to double back and retrace its steps. The points collected along the way are then sampled in a way that ensures detailed balance. The algorithm adapts ɛ in the burn‐in phase by means of a stochastic optimisation algorithm (the primal‐dual averaging). **M** is kept fixed at the identity matrix.

***AHMC***. Adaptive HMC (AHMC), proposed in Reference 65, uses Bayesian Optimisation^75^ to tune ɛ and *L*, while allowing for infinite adaptation of the parameters, with the adaptation becoming less likely as the number of iterations increases. **M** is kept fixed at the identity matrix. In AHMC, an objective function dependent on ɛ and *L* is chosen, for example, the expected squared jumping distance (ESJD) normalised by the number of leapfrog steps^65^:

(12) $$\frac{E_{p\left(.\right)}^{ɛ,L}{\left\|\theta^{\left(t+1\right)}-\theta^{\left(t\right)}\right\|}^{2}}{\sqrt{L}}.$$

The normalised ESJD is an expensive black‐box function, due to the intractable expectation with respect to the target distribution *p*(.), so it is approximated by an empirical estimator. The idea of emulation and Bayesian optimisation^76^ is adopted; the normalised ESJD is maximised by constructing a surrogate objective function using Gaussian Processes, called acquisition function. The problem is turned into the maximisation of this computationally cheap acquisition function. The same procedure can be used for the automatic tuning of the step size and the number of leapfrog steps in RMHMC or LDMC.

## 4 NOVEL STATISTICAL METHODOLOGY: COMBINATION OF MCMC WITH GAUSSIAN PROCESSES IN A DELAYED ACCEPTANCE FRAMEWORK

The application of Bayesian inference to complex physiological models poses several challenges, and we mention a few:

1. We need an efficient and accurate method for inferring the unknown parameters and quantifying their uncertainty from limited and noisy data.
2. It is expensive to obtain the data likelihood as it requires numerically integrating a system of PDEs.
3. We have a priori unknown constraints on the joint parameter space, that is, regions in the joint parameter space that violate the assumptions of the mathematical model.

***First challenge***. To address the first issue, we implement a series of state‐of‐the‐art MCMC methods (described in Section 3.2 and summarised in Table 2) and pick the method that is best in terms of accuracy and computational efficiency. Our methods include: Hamiltonian Monte Carlo, No U‐turn sampler, Riemann Manifold HMC and Lagrangian Dynamical Monte Carlo. These algorithms are combined with Bayesian optimisation for automatic performance tuning. The comparison between all of these algorithms is novel in the context of biophysical modelling.

***Second challenge***. Rasmussen addresses the second issue by combining MCMC (the HMC algorithm) with emulation using GPs as part of the GPHMC algorithm.^32^ Our contribution is to further extend and improve GPHMC.

We start by emphasising the need for GPHMC. Algorithm 1a compares the HMC algorithm (textbook knowledge) to Rasmussen's GPHMC algorithm in terms of the number of PDE evaluations required to calculate the data log likelihood and its gradient, which is included in the caption of the algorithm. Specific differences between HMC and GPHMC are marked by the text in blue colour. Algorithm 1a is just a conceptual outline; the reader is referred to Algorithms 1b and 1c for a detailed pseudocode of our extended GPHMC algorithm. Algorithm 1a in words: HMC on the true log likelihood incurs excessive computational costs due to the need to numerically solve the PDEs of the biophysical model at every leapfrog integration step of the Hamiltonian dynamics (Equation (6)) for a large number of HMC samples. The computational costs can be reduced substantially by moving the HMC scheme to the surrogate space defined by the statistical emulator. In that case, we avoid evaluating the PDEs for each of the *L* steps along the trajectory, instead we only evaluate the PDEs once per HMC sample (at the end point of the Hamiltonian trajectory) to obtain the true log likelihood, used in the decision step; the surrogate log likelihood (as predicted by the emulator) is used to guide the search along the trajectory. An HMC trajectory typically has in the order of *L* = 100–1000 steps, which if carried out in the original space would require in the order of 100 × (*d* + 1) to 1000 × (*d* + 1) (*d*: number of parameters) PDE evaluations per HMC sample, thus a reduction in the computational complexity by about two to three orders of magnitude is obtained. The term *d* + 1 is the sum of one PDE evaluation to find the log likelihood, and *d* PDE evaluations to find the numerical derivatives by a first‐order differencing scheme with respect to each of the *d* parameters. When HMC is run in the original space, several aspects regarding the calculation of the numerical derivatives of the simulated log posterior must be considered, see Appendix A.3 for details. In contrast, GPHMC requires derivatives of the GP predictive mean and variance (instead of the numerical derivatives for the simulator), which can be obtained analytically (Equations (A6) and (A7)). Additionally, for GPHMC, the smoothness, thus differentiability of the log posterior is controlled via the GP kernel (e.g., the squared exponential kernel, which is used in this study, is infinitely differentiable), while for conventional HMC, the log posterior might not be differentiable everywhere, see Appendix A.3.

Algorithm 1a **Conceptual outline for Hamiltonian Monte Carlo (HMC) versus HMC coupled with emulation using Gaussian Processes (GPHMC) algorithm. The total number of model (PDE) evaluations required for running each algorithm is: HMC – SL(d + 1) versus GPHMC – S**

1: Define a *d*‐dimensional vector ***θ*** with *θ*_*k*_ the *k*^*th*^ element, *k* = 1…*d*; S: number of HMC samples; L: number of HMC trajectory steps; *p*(**y**| ***θ***): simulator data likelihood (Equation (18))

2: **for** i = 1: S **do**.

3:  **for** j = 1: L **do**.

4:   Solve PDEs to get log*p*(**y**| ***θ***^*j*^) and $\frac{\partial \log p\left(y|\theta^{j}\right)}{\partial \theta_{k}^{j}}$

5: **end for**.

6:  Propose ***θ***^*L*^.

7:  Solve PDEs to get log*p*(**y**| ***θ***^*L*^) and accept/reject in a M‐H step.

8: **end for**.

Define a *d*‐dimensional vector ***θ*** with *θ*_*k*_ the *k*^*th*^ element, *k* = 1*d*; S: number of HMC samples; L: number of HMC trajectory steps; $\tilde{p}\left(y|\theta \right)$: emulator data likelihood (Equation (19)).

**for** i = 1: S **do**.

 
**for** j = 1: L **do**.

  Use GPs to predict $\log\tilde{p}\left(y|\theta^{j}\right)$ and $\frac{\partial \log\tilde{p}\left(y|\theta^{j}\right)}{\partial \theta_{k}^{j}}$.

** end for**

 Propose ***θ***^*L*^

 Solve PDEs to get log*p*(**y**| ***θ***^*L*^) and accept/reject in a M‐H step.

**end for**.

We use the emulator for the proposal move. The M‐H acceptance/rejection decision at the end of the trajectory is based on the true posterior distribution, the surrogate posterior distribution and the proposal probability ratio from the HMC dynamics on the emulated posterior distribution space, as shown in Equations (15) and (16). If the emulator is a poor representation of the simulator, the acceptance rate at the end of the HMC trajectory will go down, as the high probability areas in the true and the surrogate space do not match. However, the algorithm is still mathematically guaranteed to converge to the true posterior distribution, albeit at a lower convergence rate.^32^ Hence, a suboptimal emulator will not affect the mathematical accuracy of the scheme, but merely its computational efficiency.

To avoid unnecessarily solving PDEs and to potentially speed up simulations, we extend the GPHMC algorithm by introducing Delayed Acceptance^53^, ^55^ HMC, with the following idea:

***DA***. The Delayed Acceptance (DA) algorithm, proposed in Reference 53, and slightly modified by Sherlock et al.,^54^, ^55^ uses a two‐stage acceptance procedure, with two separate acceptance/rejection decisions. The idea is that the first decision is a computationally fast pre‐filter step, which upon rejection of a proposed new parameter avoids having to carry out the computationally expensive second step. As in References 54 and 55, the first acceptance probability based on the emulator is given in Equation (13) and the second acceptance probability is expressed in Equation (14):

(13) $$\alpha_{1}\left(\theta^{*}|\theta \right)\;=\;1\wedge \frac{\tilde{p}\left(\theta^{*}|y\right)q\left(\theta |\theta^{*}\right)}{\tilde{p}\left(\theta |y\right)q\left(\theta^{*}|\theta \right)},$$

(14) $$\alpha_{2}\left(\theta^{*}|\theta \right)\;=\;1\wedge \frac{p\left(\theta^{*}|y\right)}{p\left(\theta |y\right)}\frac{\tilde{p}\left(\theta |y\right)}{\tilde{p}\left(\theta^{*}|y\right)},$$

where $\tilde{p}\left(.\right)$ is the approximate posterior distribution, constructed using the emulator, *p*(.) is the exact posterior distribution, obtained using the simulator, and *q*(.) is the proposal distribution. We have used the shorthand notation *a* ∧ *b* = min{*a*, *b*}. The algorithm preserves detailed balance with respect to the posterior distribution *p*(***θ***| **y**)_._ ^53^, ^55^

For HMC‐type algorithms, Equations (13) and (14) translate to:

(15) $$\alpha_{1}^{\mathrm{HMC}}\left(\theta^{*},r^{*}|\theta ,r\right)\;=\;1\wedge \frac{\exp\left(-\tilde{H}\left[\theta^{*},r^{*}\right]\right)1}{\exp\left(-\tilde{H}\left[\theta ,r\right]\right)1}\;=\;1\wedge \frac{\exp\left(-\tilde{H}\theta^{*},r^{*}\right])}{\exp\left(-\tilde{H}\left[\theta ,r\right]\right)},$$

where $\tilde{H}\left(\theta ,r\right)=\tilde{E}\left(\theta \right)+K\left(r\right)$ is the Hamiltonian function. Also, *q*(.| .) = 1 is the proposal distribution, which comes from following a set of Hamiltonian dynamics steps, that are deterministic (i.e., the proposal is a Dirac delta function at the proposed point).

(16) $$\alpha_{2}^{\mathrm{HMC}}\left(\theta^{*},r^{*}|\theta ,r\right)\;=\;1\wedge \frac{\exp\left(-E\left(\theta^{*}\right)\right)}{\exp\left(-E\left(\theta \right)\right)}\frac{\exp\left(-\tilde{E}\left(\theta \right)\right)}{\exp\left(-\tilde{E}\left(\theta^{*}\right)\right)}.$$

The computational gain is related to the fact that we only need to carry out the computationally expensive numerical integration of the PDEs if the proposed parameter vector is accepted according to the emulator.

***Third challenge***. The third challenge in our study is addressed by constructing a GP classifier that automatically learns the infeasible parameter regions. To apply the GPHMC scheme to parameter estimation in a complex biophysical model, we typically need to deal with the fact that for certain parameter values and combinations, the underlying physical assumptions of the model are violated, or the solver used is inappropriate for the problem, producing no outputs from the simulation software. While the second matter can be tackled by for example, trying a different solver, decreasing the convergence threshold value, or increasing the number of discretisation steps, there is no clear resolution for the first issue. This may be caused by the inappropriateness of the parameter ranges, which are generally chosen by varying one parameter at a time, while fixing all the other parameters to biologically realistic values. However, when parameters are simultaneously changed, given for example, the fixed network geometry and inflow, the resulting parameter combinations may break physiological assumptions (e.g., a large arterial stiffness may not be compatible with high compliance downstream). We stress that a multivariate classifier should only be implemented upon thoroughly checking the suitability of the solver. For parameter values in the “invalid” domain, different solvers may be tried. If the crash is independent of the solver used, this suggests that the crash is of a more fundamental nature (i.e., violation of the physical model assumptions).

In a standard MCMC simulation based on the actual model, an invalid parameter vector can be assigned zero likelihood. Hence, if such a parameter vector is proposed, it will be rejected in the Metropolis‐Hastings acceptance/rejection step. However, dealing with such invalid regions in the context of emulation requires some extra care. A naive and straightforward approach is to set the likelihood for an invalid parameter vector to a very small value close to zero (i.e., the log likelihood to a negative value with large modulus) when training the GP emulator. However, this approach is unlikely to lead to a good emulator. A sudden shift to an extreme value will drive the lengthscale of the GP kernel to a very small value in the hyperparameter estimation step, which in turn will cause ripples in the GP interpolant and hence overfitting in the valid regime. We address this issue by introducing a GP classifier. Let *λ* denote a binary variable to indicate if the parameter vector ***θ*** falls into a valid regime (*λ* = 1) or invalid regime (*λ* = 0). Given a set of parameter vector ‐ label pairs obtained during the initial design and exploration phase (see below),

$$ℋ\;=\;\left\{\left(\theta_{1},\lambda_{1}\right),\ldots ,\left(\theta_{n},\lambda_{n}\right)\right\}$$

we can train a GP classifier, as reviewed in Appendix A.2.2, to predict the probability *p*(*λ* = 1| ***θ***, ℋ). We can now use these probabilities to modify the prior:

(17) $$\tilde{p}\left(\theta \right)\;=\;p\left(\theta \right)p\left(\lambda =1|\theta ,ℋ\right)/Z,$$

where *Z* =  ∫ *p*(***θ***)*p*(*λ* = 1| ***θ***, ℋ)*d****θ*** is a normalisation constant, which cancels out in the Metropolis‐Hastings acceptance/rejection step. When running in the emulated space, the sampler uses the modified prior $\tilde{p}\left(\theta \right)$ instead of the original prior *p*(***θ***) to avoid moving into invalid parameter regions (where the probability of success *p*(*λ* = 1| ***θ***, ℋ) is low).

In summary, in the exploration phase, the GP classifier is trained on the valid and invalid parameter vectors, that is, parameter vectors with (label 1) and without (label 0) software outputs. The predicted probability of failure/success is then included in the prior probability on the parameter space. In this way, moves into the invalid parameter regime are discouraged by the GP classifier, and the GP emulator does not need to provide accurate predictions here.¶ We note that a similar idea of combining a GP classifier with a GP emulator has been proposed in the context of Bayesian optimisation with unknown constraints.^77^, ^78^

***GPHMC algorithm coupled with a GP classifier and Delayed Acceptance***.

**FIGURE 2:**
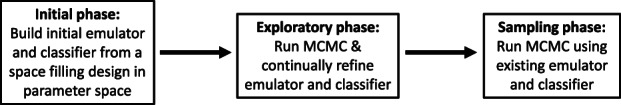
Workflow of the GPHMC algorithm.^32^ The emulator and classifier constructed in the initial phase are continually refined as HMC is run in the exploratory phase. HMC in the sampling phase proceeds by drawing samples from the asymptotically exact posterior distribution, with the use of the emulator and classifier, which are no longer updated

**FIGURE 3:**
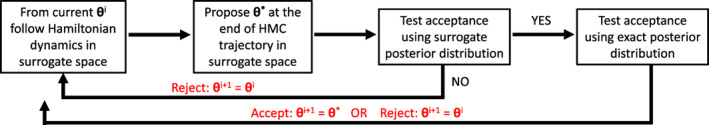
Workflow of our proposed Delayed Acceptance within GPHMC^32^ method

We now put all the elements explained above together. Thus, we provide a description of the implementation of the DA‐GPHMC algorithm, with our adaptation to mathematical models with limited valid domains of a priori unknown location. Pseudocode for GPHMC coupled with a GP classifier and DA can be found in Algorithms 1b and 1c. Specific equations used in the practical implementation of the algorithm are given in the Simulations Section 5. A diagram summarising Rasmussen's GPHMC algorithm can be seen in Figure 2, and in Figure 3 we illustrate the workflow of our proposed DA‐GPHMC method, where the latter algorithm is used within the former.

- *Initial design stage*. Starting from a space filling design in parameter space to capture the compact support of the biophysical parameters (see Section 2.1 for more details), for example, using a Latin hypercube^79^ or a Sobol sequence,^80^ integrate the PDEs numerically for each parameter vector to get the true log likelihood of the physiological data given the PDE parameters, as well as the success labels. Use these points to build a GP emulator and a GP classifier. Only those parameter vectors which yield successful PDE simulations are added to the list of training points for the GP regression model. All points regardless of whether or not they provide a successful simulation are added to the list of training points for the GP classifier, to enable the classifier to learn the infeasible regions that break the biophysical assumptions.
- Exploratory phase. Gather information about the target distribution by running HMC on the surrogate log posterior of the PDE parameters; the end point of every HMC trajectory is subject to a 2‐stage DA Metropolis‐Hastings accept/reject step (see Equations (15) and (16)), for which the simulator is called. The emulator and the classifier are sequentially refined (i.e., optimum covariance hyperparameters are found by maximisation of the log marginal likelihood of the GP training points – see Equation (A2)) as new points are accepted. Accepted parameter vectors are iteratively added as further training points to those used in the initial design stage. The points in the initial design stage are gradually removed from the list of training points as they tend to come from low posterior density regions and can bias the inference results – see lines 16 and 27 in Algorithm 1b. As HMC explores the parameter space, it gets closer to the equilibrium distribution (burn‐in phase). This ensures the algorithm sequentially zooms into the regions of high posterior probability. Following,^32^ we set the emulated “potential energy” of the HMC algorithm (see Section 3.2) to

$$\tilde{E}\left(\theta^{*}\right)=\frac{E\left(f\left(\theta^{*}\right)|D\right)-\sqrt{\mathrm{var}\left(f\left(\theta^{*}\right)|D\right)}}{2\sigma^{2}}+\frac{n}{2}\log\left(2{\pi \sigma }^{2}\right)-\log\tilde{p}\left(\theta^{*}\right).$$

Here *σ*^2^ is the variance of the errors, $\tilde{p}\left(\theta^{*}\right)$ is the prior distribution (Equation (17)), *f*(.) is the emulated residual sum‐of‐squares function, $E\left(f\left(\theta^{*}\right)|D\right)$ is the GP posterior predictive mean given the training points *D* (see Equation (A4)) and $\sqrt{\mathrm{var}\left(f\left(\theta^{*}\right)|D\right)}$ is the GP posterior predictive standard deviation (see Equation (A5)) for the residual sum‐of‐squares of the physiological data at unseen parameter configurations θ^*^ conditional on the training points *D*. This drives the exploration into regions with high posterior probability (low value of $E\left(.\right)$) or high uncertainty (large value of $\sqrt{\mathrm{var}\left(.\right)}$). If $\sqrt{\mathrm{var}\left(.\right)}$>3 along the trajectory, the simulation is stopped prematurely before reaching the end of the trajectory, as the algorithm steps into a region of high uncertainty, where the GP needs to be further trained. The log likelihood is computed at this point by numerically solving the PDEs of the biophysical model, and the corresponding success label is obtained. We note, as an aside, that this follows the same exploitation‐exploration trade‐off principle as in optimistic acquisition functions used for Bayesian optimisation, see sec. IV in Reference 76.

- *Sampling phase*. Use the emulator and the classifier constructed in the exploratory phase to draw samples from the target distribution using HMC, or any of its variants described in Section 3.2. At this stage, the emulator and the classifier are no longer updated. The emulated “potential energy” in the HMC algorithm is set to

$$\tilde{E}\left(\theta^{*}\right)=\frac{E\left(f\left(\theta^{*}\right)|D\right)}{2\sigma^{2}}+\frac{n}{2}\log\left(2{\pi \sigma }^{2}\right)-\log\tilde{p}\left(\theta^{*}\right).$$

Note that the numerator in the first term is the expected sum‐of‐squares error, which combined with the normalisation term in the middle gives the log likelihood of the data, and the final term is the log prior. The end point of the trajectory is subject to a 2‐stage DA Metropolis‐Hastings accept/reject step (see Equations (15) and (16)), based on the simulator. The rejection rate is monitored, and this indicates how well the GP emulator has captured the log posterior density. A large number of rejections calls for an extension of the exploratory phase.

The surrogate log likelihood is used throughout the trajectory, and the simulator (true) log likelihood is used in the final accept/reject step, which implies that asymptotically the samples are drawn from the exact target distribution. The accuracy of the emulator can be checked by diagnostics,^81^ for example, GP predictions for the log likelihood corresponding to unseen (test) inputs should be close to the true (simulator) log likelihood values, that is, these points should lie on the equality line when plotted against each other. In our analysis, we employ HMC in the exploratory phase. For the sampling phase we employ the algorithms described above: NUTS, Adaptive HMC, Adaptive RMHMC, Adaptive LDMC (the adaptation of the tuning parameters is performed with Bayesian optimisation). Note that in the GPHMC algorithm, when we need to calculate derivatives of the emulated log posterior, we use the fact that GPs are closed under differentiation, that is, the derivatives of a GP are also GPs (though with different covariance structures), provided the kernel is differentiable. See sec. 9.4 in Reference 74 for further details.

Algorithm 1b **GP HMC algorithm with DA – initial and exploratory phase**

1: Let $D=\left\{\left(X_{e},S_{e}\right):\lambda =1\right\}$: training set for the GP emulator and ℋ = (**X**, **λ**): training set for the GP classifier, where **X**: *n* × *d* matrix of input parameter vectors ***θ***, and **X**_*e*_: subset of **X**, $S_{e}$: vector of residual sum‐of‐square (RSS) values, and **λ**: *n* × 1 vector of success labels (0,1). Denote *S*: number of HMC samples, L: number of HMC trajectory steps, ɛ: step size, $S\left(\theta \right)$: simulator RSS, $\tilde{S}\left(\theta \right)$: emulator RSS, **M**: mass matrix, *f*(.): emulated RSS function, and *p*(**y**| ***θ***): simulator data likelihood, $\tilde{p}\left(y|\theta \right)$: emulator data likelihood, *E*(***θ***): true potential function, $\tilde{E}\left(\theta \right)$: surrogate potential function, *K*(**r**): kinetic energy for the momentum variable **r**.

2: $\log p\left(y|\theta \right)=-\frac{S\left(\theta \right)}{2\sigma^{2}}-\frac{n}{2}\log\left(2{\pi \sigma }^{2}\right)$ and $\log\tilde{p}\left(y|\theta \right)=-\frac{\tilde{S}\left(\theta \right)}{2\sigma^{2}}-\frac{n}{2}\log\left(2{\pi \sigma }^{2}\right)$ for $ɛ\underset{∼}{\mathrm{iid}}ℳ\mathrm{VN}\left(0,\sigma^{2}I\right)$; *E*(***θ***) =  − (log*p*(**y**| ***θ***) + log*p*(***θ***)), where *p*(***θ***): prior distribution; $\tilde{E}\left(\theta \right)=-\left(\log\tilde{p}\left(y|\theta \right)+\log\tilde{p}\left(\theta \right)\right)$, where $\tilde{p}\left(\theta \right)$: modified prior distribution (see Equation (17)).

3: **INITIAL DESIGN STAGE**: Build the GP emulator and the GP classifier.

4: **EXPLORATORY PHASE**: Set ***θ***^0^ and *l* = 1, where *l* marks the *l*^*th*^ point being deleted.

5: **for** i = 1: S **do** ▷ loop over HMC samples

6:  Draw **r** ∼ exp(−*K*(**r**)) and let ***θ***_0_ = ***θ***^*i* − 1^ and ${\left.r_{0}=r+\frac{ɛ}{2}\frac{\partial \tilde{E}}{\partial \theta }\right|}_{\theta_{0}}$

7:  **for** j = 1: L **do** ▷ loop over HMC steps

8:   ***θ***_*j*_ = ***θ***_*j* − 1_ + ɛ**M**^−1^**r**_*j* − 1_; ${\left.r_{j}=r_{j-1}+ɛ\frac{\partial \tilde{E}}{\partial \theta }\right|}_{\theta_{j}}$

9:  **end for**

10:  ${\left.r_{L}=r_{L-1}+\frac{ɛ}{2}\frac{\partial \tilde{E}}{\partial \theta }\right|}_{\theta_{L}}$

11:  Set proposed points (θ^*^, r^*^) = (***θ***_*L*_, **r**_*L*_) and $\tilde{S}\left(\theta^{*}\right)=E\left(f\left(\theta^{*}\right)|D\right)-\sqrt{\mathrm{var}\left(f\left(\theta^{*}\right)|D\right)}$, where $E\left(f\left(\theta^{*}\right)|D\right)$ and $\sqrt{\mathrm{var}\left(f\left(\theta^{*}\right)|D\right)}$ are the GP posterior predictive mean and standard deviation (Equations (A4) and (A5))

12:  M‐H accept/reject step with 1st stage acceptance probability (emulator based): $\alpha_{1}\left(\theta^{*},r^{*}|\theta^{i-1},r\right)\;=\;1\wedge \frac{\exp\left(-\tilde{E}\left(\theta^{*}\right)\right)}{\exp\left(-\tilde{E}\left(\theta^{i-1}\right)\right)}\frac{\exp\left(-K\left(r^{*}\right)\right)}{\exp\left(-K\left(r\right)\right)},$ with $\tilde{E}$ computed from $\tilde{S}$ as explained in line 2.

13:  **if** *α*_1_ ≥ *v*_1_, *v*_1_ ∼ *U*(0, 1) **then**, solve PDEs for θ^*^ = ***θ***_*L*_ to get $\left(\theta^{*},S\left(\theta^{*}\right),\lambda^{*}\right)$ ‐ see Sections 4 and 5.

14: Calculate the 2nd stage acceptance probability (simulator based): $\alpha_{2}\left(\theta^{*}|\theta^{i-1}\right)\;=\;1\wedge \frac{\exp\left(-E\left(\theta^{*}\right)\right)}{\exp\left(-E\left(\theta^{i-1}\right)\right)}\frac{\exp\left(-\tilde{E}\left(\theta^{i-1}\right)\right)}{\exp\left(-\tilde{E}\left(\theta^{*}\right)\right)},$ with *E* computed from S as explained in line 2

15:   **if** *α*_2_ ≥ *v*_2_, *v*_2_ ∼ *U*(0, 1) **then** set ***θ***^*i*^ = θ^*^.

16:    **if** $S\left(\theta^{l}\right)>T$, where *T*: threshold value chosen based on the S values from the initial design stage (e.g. *T* = 10th percentile) **then** update $D=D\backslash \left(\theta^{l},S\left(\theta^{l}\right)\right),\;ℋ=ℋ\backslash \left(\theta^{l},\lambda^{l}\right)$.

17:    **else** set l = l + 1.

18: **   end if**.

19:    Re‐train GP emulator with $D=D\cup \left(\theta^{*},S\left(\theta^{*}\right)\right)$ and GP classifier with ℋ = ℋ ∪ (θ^*^, λ^*^)

20:   **else**

21:    ***θ***^*i*^ = ***θ***^*i* − 1^

22:   **end if**.

23:  **else**

24:   ***θ***^*i*^ = ***θ***^*i* − 1^

25:  **end if**.

26: **end for**.

27: Update $D=D\backslash \left(\theta ,S\left(\theta \right)\right),\;ℋ=ℋ\backslash \left(\theta ,\lambda \right)$ for the remaining ***θ*** for which $S\left(\theta \right)>T$.

28: Re‐train GP emulator with new *D* and GP classifier with new ℋ and enter the **SAMPLING PHASE**.

Algorithm 1c **GP HMC algorithm with DA – sampling phase**

1: Denote ***θ***: input parameter vector, *S*: number of HMC samples, L: number of HMC trajectory steps, ɛ: step size, $S\left(\theta \right)$: simulator residual sum‐of‐square (RSS) value, $\tilde{S}\left(\theta \right)$: emulator RSS, **M**: mass matrix, *f*(.): emulated RSS function, *p*(**y**| ***θ***): simulator data likelihood, $\tilde{p}\left(y|\theta \right)$: emulator data likelihood, *E*(***θ***): true potential function, $\tilde{E}\left(\theta \right)$: surrogate potential function, *K*(**r**): kinetic energy for the momentum variable **r**.

2: $\log p\left(y|\theta \right)=-\frac{S\left(\theta \right)}{2\sigma^{2}}-\frac{n}{2}\log\left(2{\pi \sigma }^{2}\right)$ and $\log\tilde{p}\left(y|\theta \right)=-\frac{\tilde{S}\left(\theta \right)}{2\sigma^{2}}-\frac{n}{2}\log\left(2{\pi \sigma }^{2}\right)$ for $ɛ\underset{∼}{\mathrm{iid}}ℳ\mathrm{VN}\left(0,\sigma^{2}I\right)$; *E*(***θ***) =  − (log*p*(**y**| ***θ***) + log*p*(***θ***)), where *p*(***θ***): prior distribution; $\tilde{E}\left(\theta \right)=-\left(\log\tilde{p}\left(y|\theta \right)+\log\tilde{p}\left(\theta \right)\right)$, where $\tilde{p}\left(\theta \right)$: modified prior distribution (see Equation (17)).

3: Initialise ***θ***^0^.

4: **for** i = 1: S **do**                           ▷ loop over HMC samples

5:  Draw **r** ∼ exp(−*K*(**r**)).

6:  Let ***θ***_0_ = ***θ***^*i* − 1^ and ${\left.r_{0}=r+\frac{ɛ}{2}\frac{\partial \tilde{E}}{\partial \theta }\right|}_{\theta_{0}}$.

7:  **for** j = 1: L **do**                           ▷loop over HMC steps

8:   ***θ***_*j*_ = ***θ***_*j* − 1_ + ɛ**M**^−1^**r**_*j* − 1_

9:    ${\left.r_{j}=r_{j-1}+ɛ\frac{\partial \tilde{E}}{\partial \theta }\right|}_{\theta_{j}}$

10:  **end for**

11:   ${\left.r_{L}=r_{L-1}+\frac{ɛ}{2}\frac{\partial \tilde{E}}{\partial \theta }\right|}_{\theta_{L}}$

12:  Set proposed points (θ^*^, r^*^) = (***θ***_*L*_, **r**_*L*_) and $\tilde{S}\left(\theta^{*}\right)=Ε\left(f\left(\theta^{*}\right)|D\right)$, where Ε(*f*(θ^*^)| *D*)is the GP posterior predictive mean (see Equation (A4))

13:  Compute $\tilde{E}$ from $\tilde{S}$ as explained in line 2.

14:  M‐H accept/reject step with 1st stage acceptance probability (emulator based):

$$\alpha_{1}\left(\theta^{*},r^{*}|\theta^{i-1},r\right)\;=\;1\wedge \frac{\exp\left(-\tilde{E}\left(\theta^{*}\right)\right)}{\exp\left(-\tilde{E}\left(\theta^{i-1}\right)\right)}\frac{\exp\left(-K\left(r^{*}\right)\right)}{\exp\left(-K\left(r\right)\right)}$$

15:  **if** *α*_1_ ≥ *v*_1_, *v*_1_ ∼ *U*(0, 1) **then**.

16:   Solve PDEs for θ^*^ to get $\left(\theta^{*},S\left(\theta^{*}\right),\lambda^{*}\right)$ – see Sections 4 and 5.

17:   Compute *E* from S as explained in line 2.

18:   Calculate the 2nd stage acceptance probability (simulator based):

$$\alpha_{2}\left(\theta^{*}|\theta^{i-1}\right)\;=\;1\wedge \frac{\exp\left(-E\left(\theta^{*}\right)\right)}{\exp\left(-E\left(\theta^{i-1}\right)\right)}\frac{\exp\left(-\tilde{E}\left(\theta^{i-1}\right)\right)}{\exp\left(-\tilde{E}\left(\theta^{*}\right)\right)}$$

19:   **if** *α*_2_ ≥ *v*_2_, *v*_2_ ∼ *U*(0, 1) **then**.

20:    Set ***θ***^*i*^ = θ^*^

21:   **else**

22:    ***θ***^*i*^ = ***θ***^*i* − 1^

23:   **end if**.

24:  **else**

25:   ***θ***^*i*^ = ***θ***^*i* − 1^

26:  **end if**.

27: **end for**.

## 5 SIMULATIONS

### 5.1 Software

Simulations are run on a RedHat Enterprise Linux 6 machine with Intel(R) Xeon(R) CPU E5‐2680 v2 2.80GHz and 32GB RAM. Our code uses Matlab and implements the GP models using the GPstuff toolbox.^82^ To run the No U‐turn sampler, Riemann Manifold HMC and Lagrangian Dynamical MC, we used the Matlab implementations developed by the authors of the papers where these algorithms were proposed, and they are available as follows.^62^, ^63^, ^64^ The simulated pressure waveforms are obtained by numerically integrating the PDEs described in Section 2.1 with a two step Lax‐Wendroff Scheme^83^ implemented in C++ by Olufsen et al.^6^ The numerical integration of the PDEs to obtain one single model solution requires 1 s CPU time and 24 s elapsed time on our hardware.

Our software can be found at: https://github.com/LMihaelaP/BayesianUQ_CardiovascularModelling.git.

### 5.2 Method implementation details

#### 5.2.1 GP compact support

The emulator needs compact support. The bounds for the parameters were taken to be biologically meaningful, as indicated in Table 1.

#### 5.2.2 GP models

In our analysis, for GP regression, that is, for building the emulator, we use a squared exponential kernel (see sec. 4.2 in Reference 74) for the covariance function, as chosen by cross‐validation based on the data. Additionally, for the GP classifier a Matérn 3/2 kernel (see sec. 4.2 in Reference 74) is used, which captures the classification boundaries, which are assumed not very smooth.** We approximate the intractable integral in Equation (A12), which is required for obtaining the class probabilities, with expectation propagation; see sec. 3.6 in Reference 74 for details.

#### 5.2.3 GP analytical derivatives

For HMC we require the first order derivatives of the GP posterior predictive mean and variance – see Equations (A6) and (A7), while for RMHMC and LDMC we need the second and third order derivatives, which we obtain analytically,†† since differentiation is a linear operator, so the derivative of a GP is again a GP for differentiable kernels; see sec. 9.4 in Reference 74.

#### 5.2.4 GPHMC phases

***Initial design stage***. The first design stage of GPHMC comprises 600 PDE evaluations for parameter configurations generated from a Sobol sequence, which helps us build the initial GP emulator and classifier. The number of PDE evaluations is chosen to ensure a certain accuracy of the emulator, determined by the efficiency of the MCMC sampler in the beginning of the exploratory phase (the acceptance rate of the sampler running on the initial emulator should not be too low (e.g., below 20%).

***Exploratory phase***. In the exploratory phase of GPHMC^32^ we run HMC for 1000 iterations. We tried to ensure that a minimum number of training points is stored (as to boost computational efficiency), while preserving a high enough emulator accuracy (as quantified by GP diagnostics^81^), and a high acceptance rate in the sampling phase (>65%^22^). The samples collected in the exploratory phase are used to refine the GP models for regression and classification (i.e., update the covariance hyperparameters by re‐optimising the log marginal likelihood of the GP training points – see Equation (A2)) and to get closer to the equilibrium distribution. The emulator to be used in the sampling phase consists of 400 training points.

***Sampling phase***. We apply the GPHMC scheme described in Section 4, with the following algorithms in the sampling phase:

- No U‐turn sampler algorithm,
- Adaptive Hamiltonian Monte Carlo (AHMC) with Bayesian optimisation for performance tuning,
- Adaptive Riemann Manifold Hamiltonian Monte Carlo (ARMHMC) with Bayesian optimisation for performance tuning,
- Adaptive Lagrangian Dynamical Monte Carlo (ALDMC) algorithm with Bayesian optimisation for performance tuning.

The performance tuning involves setting the integration step size and the number of integration steps between subsequent Metroplis‐Hastings acceptance steps.

In the sampling phase of GPHMC^32^ we allow for further 500 samples as burn‐in phase, and 5000 samples are subsequently drawn and used for inference. The chosen number of burn‐in steps ensures that the value for the multivariate potential scale reduction factor (MPSRF) falls below the standard threshold of 1.1, MPSRF ≤ 1.1_,_ ^84^ which is taken as an indication of sufficient convergence.

#### 5.2.5 Bayesian optimisation for HMC performance tuning

The adaptive algorithms with Bayesian optimisation^65^ require us to construct an initial GP for the normalised squared jumping distance in Equation (12), as a function of the step size and the number of leapfrog steps, which need compact support. For the adaptive algorithms, we use the following ranges for the step size and the number of leapfrog steps: for HMC, *L* ∈ {1, …, 50}, ɛ ∈ [10^−4^, 5 × 10^−3^], and for RMHMC and LDMC, *L* ∈ {1, …, 40}, ɛ ∈ [10^−2^, 8 × 10^−2^]. These ranges are chosen in a way that ensures a high effective sample size (ESS),^85^ while no anticorrelation is induced (which would make ESS greater than the total number of MCMC samples).^86^ The initial GP for the normalised squared jumping distance is constructed based on 20 (ɛ, *L*) parameter vectors, and for each parameter vector we obtain 10 MCMC samples for the PDE parameters to estimate the expectation in Equation (12).

#### 5.2.6 MCMC convergence with MPSRF

Every algorithm in the sampling phase is run 10 times from different random number generator seeds and different starting values for the parameters, selected from the points collected in the exploratory phase, to make it less likely that we start in a low probability region. In choosing the number of independent chains, we followed the suggestion given in the study by Cowles et al.^87^ The authors advise to use 10 independent chains if the posterior distribution is unimodal, and more for multi‐modal distributions. Generally, as proposed in the study by Gelman and Rubin,^88^ an optimisation, mode‐finding algorithm may be used to find regions of high density, and the starting values can be generated by sampling from a mixture of t‐distributions centred at these modes.

#### 5.2.7 MCMC mixing with ESS

For each of the 10 simulations, we record the minimum ESS across all parameters (i.e., compute ESS for each of the four parameters, and take the minimum from this set of 4, see Equation (20)), the minimum ESS divided by the number of forward (PDE) simulations, and the minimum ESS divided by the total CPU time for the entire simulation.

#### 5.2.8 MCMC set‐up

We apply all the algorithms to the pulmonary circulation model, to carry out a comparative assessment of their performance. The data used (pulmonary blood pressure) are described in Sections 2.3 and 2.4. The four biophysical parameters that are to be learned, given in Equation (5), are the vessel stiffness and the Windkessel parameters.

We assume the following:

• Likelihood based on the simulator: $y_{i}|\theta ∼N\left(m_{i}\left(\theta \right),\sigma^{2}\right)$, that is,

(18) $$p\left(y|\theta ,\sigma^{2}\right)={\left(\frac{1}{\sqrt{2{\pi \sigma }^{2}}}\right)}^{n}\exp\left(-\frac{\sum_{i=1}^{n}{\left(y_{i}-m_{i}\left(\theta \right)\right)}^{2}}{2\sigma^{2}}\right),$$

where **m**(***θ***) = (*m*_1_(***θ***), …, *m*_*n*_(***θ***)) is the vector of predictions from the PDEs of Equations (1), (2), (3)), **y** = (*y*_1_, …, *y*_*n*_) is the vector of measured data (pressure data), and *n* is the number of time points. If a PDE simulation is unsuccessful, we set the likelihood to zero in the MCMC simulation, that is, *p*(**y**| ***θ***, *σ*^2^) = 0.

• Likelihood based on the emulator:

(19) $$\tilde{p}\left(y|\theta ,\sigma^{2}\right)={\left(\frac{1}{\sqrt{2{\pi \sigma }^{2}}}\right)}^{n}\exp\left(-\frac{\tilde{S}\left(\theta \right)}{2\sigma^{2}}\right),$$

where $\tilde{S}\left(\theta \right)$ is the value of the residual sum‐of‐squares $\sum_{i=1}^{n}{\left(y_{i}-m_{i}\left(\theta \right)\right)}^{2}$ predicted by the emulator (the GP posterior predictive mean – Equation (A4)) for the particular ***θ***. The covariance hyperparameters are refined by optimisation of the log marginal likelihood for the GP regression (see Equation (A2)) and classification (see Equation (A12)) at every step in the exploratory phase of the GPHMC algorithm.^32^ The emulator and classifier thus created are used in the sampling phase, with no further updates.

• Priors: We use a rescaled Beta(1, 1) distribution (to ensure positive support for the biophysical parameters) with support within physiologically realistic ranges (given in Table 1), *θ*_*i*_ ∼ Beta(1, 1), *l*_*i*_ ≤ *θ*_*i*_ ≤ *u*_*i*_, where *i* = 1, …, *d*, with *d* = 4 being the parameter dimensionality. For the emulation phase, this prior is modified by the GP classifier according to Equation (17).

For the noise variance *σ*^2^, we follow our previous work in Reference 7, and use a conjugate weakly informative inverse gamma prior, *σ*^2^ ∼ Inv ‐ Gamma(*a*, *b*), with *a* = 0.5*n*_*s*_ and $b=0.5n_{s}\gamma_{s}^{2}$, where $\gamma_{s}^{2}=\frac{\min\left(\sum_{i=1}^{n}{\left(y_{i}-m_{i}\left(\theta \right)\right)}^{2}\right)}{n-d}$ is the prior value for *σ*^2^ and *n*_*s*_ = 1 is the prior accuracy for $\gamma_{s}^{2}$:

$$p\left(\sigma^{2}\right)=\frac{b^{a}}{\Gamma \left(a\right)}{\left(\sigma^{2}\right)}^{-a-1}\exp\left(-\frac{b}{\sigma^{2}}\right).$$

• Posterior distribution:

$$p\left(\theta ,\sigma^{2}|y\right)\propto p\left(y|\theta ,\sigma^{2}\right)p\left(\theta \right)p\left(\sigma^{2}\right),$$

and

$$\tilde{p}\left(\theta ,\sigma^{2}|y\right)\propto \tilde{p}\left(y|\theta ,\sigma^{2}\right)\tilde{p}\left(\theta \right)p\left(\sigma^{2}\right).$$

The parameters ***θ*** are sampled using all the algorithms tested. At every MCMC iteration, given the value for ***θ***, *σ*^2^ is sampled with a Gibbs sampling step from *p*(*σ*^2^| ***θ***, **y**), which due to the conjugacy of the prior is available in closed‐form:

$$p\left(\sigma^{2}|\theta ,y\right)=\mathrm{Inv}‐\text{Gamma}\left(\frac{n}{2}+a,0.5\sum_{i=1}^{n}{\left(y_{i}-m_{i}\left(\theta \right)\right)}^{2}+b\right).$$

We keep *σ*^2^ fixed in the burn‐in phase and infer it in the sampling phase.

#### 5.2.9 Parameter transformations

To improve numerical stability and reduce round‐off errors,^7^ the original parameters *θ*_*i*_ are scaled in the order of 1 for building the GP emulator and GP classifier: $\theta_{i}\in \left[l_{i},u_{i}\right]\to \frac{\theta_{i}}{s_{i}}\in \left[-1,1\right]$, where *s*_*i*_ is a scaling factor which ensures $\frac{\theta_{i}}{s_{i}}\in \left[-1,1\right]$. In addition, the HMC algorithm requires unbounded parameters: $\theta_{i}\in \left[l_{i},u_{i}\right]\to \log_{e}\frac{\theta_{i}-l_{i}}{u_{i}-\theta_{i}}\in I\;R$. The transformed parameters are then mapped back via the inverse transformation into the original domain *θ*_*i*_ ∈ [*l*_*i*_, *u*_*i*_] for the PDE simulator, that is, when they are inserted into the mathematical PDE model described in Section 2.1.

## 6 NUMERICAL RESULTS

### 6.1 Physiological data

#### 6.1.1 Accuracy

**FIGURE 4:**
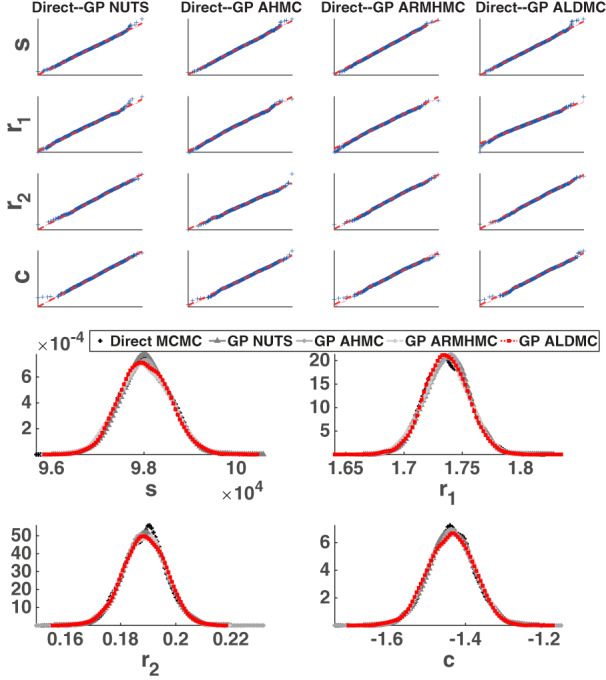
Measured (physiological) data inference results. Top panel: QQ plots, where we plot the marginal posterior densities obtained with every emulation algorithm against the marginal posterior densities obtained with the long MCMC reference run based on the actual PDE model rather than the emulator for the four parameters (*s*: stiffness, *r*_1_, *r*_2_, *c*: Windkessel parameters, see Equation (5)). Bottom panel: Kernel density estimation plots for the marginal posterior probability densities of the four parameters (*s*: stiffness, *r*_1_, *r*_2_, *c*: Windkessel parameters, see Equation (5)) for the GP emulation algorithms and the direct MCMC algorithm. To obtain the marginal posterior density, we have used the kernel smoothing function estimate for univariate data with the optimal bandwidth for normal densities.^90^ Samples were drawn using the MCMC algorithms from Section 3.2, which were combined with the GP emulator and classifier as described in Section 4. The Adaptive Metropolis^89^ algorithm was used to draw samples approximately from the exact posterior distribution. This enables to test if the emulation approach gives any bias. The data used for this study are measured (physiological) pulmonary pressure time series described in Section 2.3, for which the true parameter values are unknown. Each simulation for the emulation algorithms consisted of 5000 sampling phase draws and 500 burn‐in draws (the latter were discarded), while for the direct MCMC algorithm, the simulation consisted of 150,000 sampling phase draws and 500 burn‐in draws, which were discarded. Legend: GP: Gaussian Process; NUTS: No U‐turn Sampler; AHMC: Adaptive Hamiltonian Monte Carlo; ARMHMC: Adaptive Riemann Manifold Hamiltonian Monte Carlo; ALDMC: Adaptive Lagrangian Dynamical Monte Carlo

**FIGURE A1:**
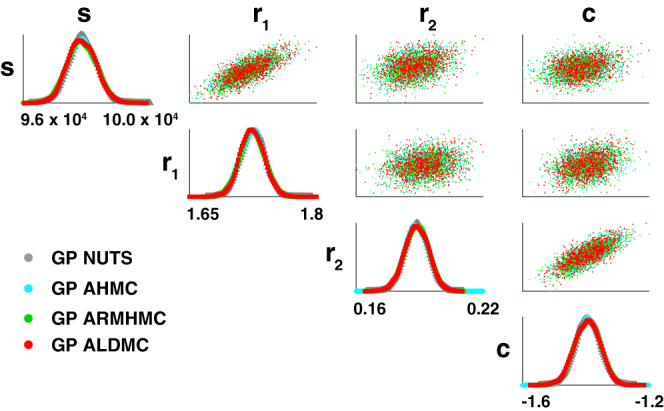
Measured (physiological) data inference results. Marginal posterior densities obtained with kernel density estimation, as well as the pairwise scatterplots, based on the posterior samples drawn using all emulation Gaussian Process Hamiltonian and Lagrangian Dynamical Monte Carlo algorithms. Each simulation consisted of 5000 sampling phase draws and 500 burn‐in draws (the latter were discarded). Legend: GP: Gaussian Process; NUTS: No U‐turn Sampler; AHMC: Adaptive Hamiltonian Monte Carlo; ARMHMC: Adaptive Riemann Manifold Hamiltonian Monte Carlo; ALDMC: Adaptive Lagrangian Dynamical Monte Carlo

In Figure 4 (bottom panel) we superimpose kernel density estimation plots for the marginal posterior densities of the PDE parameters ***θ*** = (*s*, *r*_1_, *r*_2_, *c*), obtained with the various emulation HMC algorithms compared in our study. To test whether the emulation approach gives any bias in the results, we also present results obtained with a long‐run MCMC sampler (the Adaptive Metropolis [AM] algorithm^89^) that draws samples directly from the asymptotically exact posterior distribution, which we take as a proxy for the gold standard. HMC running directly on the original posterior distribution would incur excessive computational costs (see Section 4 for a discussion on this), hence in our work we opt for a random‐walk algorithm (AM). To obtain the marginal posterior densities, we have used the kernel smoothing function estimate for univariate data with the optimal bandwidth for normal densities.^90^ We have also constructed QQ plots (see the top panel of Figure 4), where we plot the marginal posterior densities from the direct AM algorithm against any emulation HMC algorithm for the four parameters. The kernel density plots show overlapping densities for the different algorithms, and the QQ plot suggests that the algorithms provide samples from approximately the same density. In addition, in Figure A1 of the Appendix we superimpose the pairwise scatterplots for all methods: GP AHMC, GP NUTS, GP ARMHMC and GP ALDMC. We notice great overlap between the pairwise points for all algorithms, indicating agreement between the methods in input space. Therefore, given that all the densities predicted by the different sampling algorithms tend to agree and are very similar, and there is no indication of lack of convergence (as seen from Table 3), quantified using MPSRF^84^ (MPSRF ≤1.1), we have strong evidence that all sampling algorithms included in our study converge to the true posterior distribution, and no bias is introduced by the emulator.

**TABLE 3:** A comparison of the various MCMC algorithms, reviewed in Section 3.2, tested on the measured (physiological) pulmonary blood pressure time series described in Section 2.3

Algorithm	Pre‐processing: CPU time (s) and no. PDEs	Sampling: CPU time (s) and no. PDEs	$\frac{\mathrm{Min}\left(\mathrm{ESS}\right)}{N}$	$\frac{\mathrm{Min}\left(\mathrm{ESS}\right)}{\text{CPUtime}}$	$\frac{\mathrm{Min}\left(\mathrm{ESS}\right)}{\mathrm{no}.\text{PDEs}}$	Acceptance probability	MPSRF
GP NUTS	0 and 0	6025 (87) and 4841 (10)	0.21 (0.02)	0.18 (0.01)	0.22 (0.02)	0.97 (0.002)	1.001
GP AHMC	539 and 198	8908 (362) and 4929 (11)	0.29 (0.05)	0.16 (0.02)	0.30 (0.05)	0.98 (0.003)	1.001
GP ARMHMC	2102 and 199	35,390 (1675) and 4997 (2)	0.61 (0.12)	0.09 (0.02)	0.60 (0.12)	0.99 (0.001)	1.001
GP ALDMC	499 and 200	7570 (509) and 4996 (2)	0.65 (0.14)	0.43 (0.08)	0.65 (0.14)	0.99 (0.001)	1.000

*Note:* These were carried out in the sampling phase of the GPHMC algorithm described in Section 4. Ten chains with 5000 sampling phase draws and 500 burn‐in draws (which were discarded) were run. The mean and the standard deviation in brackets is provided for the minimum effective sample size normalised by the total number of MCMC samples *N*, across all four parameters (see Equation (20)), and the mean is obtained over all 10 simulations. We also provide min(ESS) normalised by CPU time, min(ESS) normalised by the number of forward (PDE) evaluations, the acceptance probability, the multivariate potential scale reduction factor (MPSRF), as well as the pre‐processing CPU times and number of forward evaluations, and the sampling phase CPU times and number of forward evaluations. For the adaptive algorithms, 200 MCMC samples are drawn and used for pre‐processing, needed for building an initial GP for the normalised squared jumping distance, which is used in the Bayesian optimisation; NUTS does not require any pre‐processing. Legend: GP: Gaussian Process; NUTS: No U‐turn Sampler; AHMC: Adaptive Hamiltonian Monte Carlo; ARMHMC: Adaptive Riemann Manifold Hamiltonian Monte Carlo; ALDMC: Adaptive Lagrangian Dynamical Monte Carlo.

#### 6.1.2 Model fits

**FIGURE 5:**
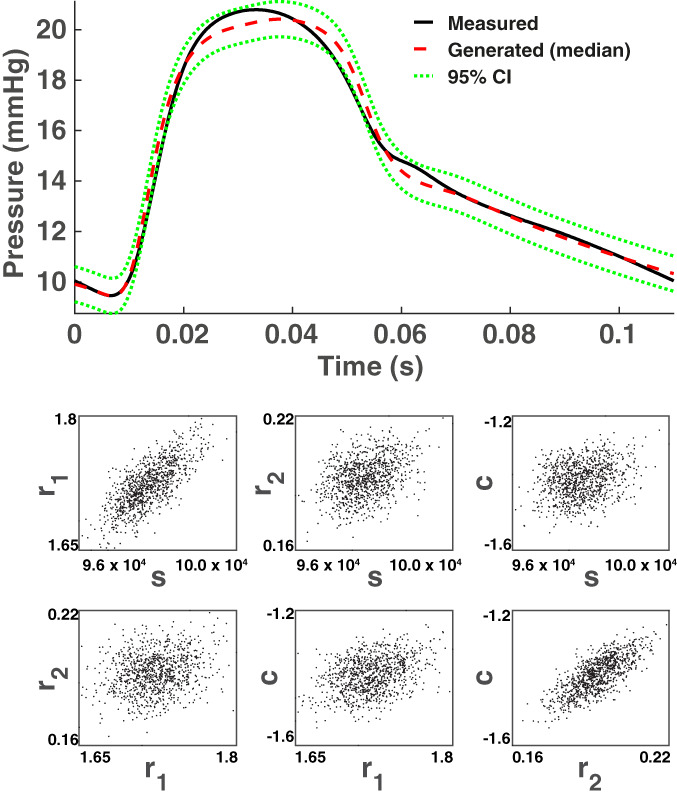
Top panel: Measured (physiological) blood pressure time series (black solid line) compared with simulated pressure time series (red dashed line), which is the posterior median signal obtained from the simulations with the Gaussian Process Adaptive Lagrangian Dynamical Monte Carlo sampler (GP ALDMC). With green dotted lines we show the 95% predictive credible interval, calculated analytically as *m*(***θ***) ± 1.96*σ*_total_, where *m*(***θ***) is the mean posterior pressure signal, and $\sigma_{\text{total}}=\sqrt{\sigma_{\text{error}}^{2}+\sigma_{\text{signal}}^{2}}$, with $\sigma_{\text{error}}^{2}$ being the mean posterior estimate for the noise variance, and $\sigma_{\text{signal}}^{2}$ being the variance of the distribution of the signals, which are model predictions obtained with the MCMC posterior parameter samples. Bottom panel: Parameter correlations obtained from the posterior samples based on the measured data

**FIGURE A2:**
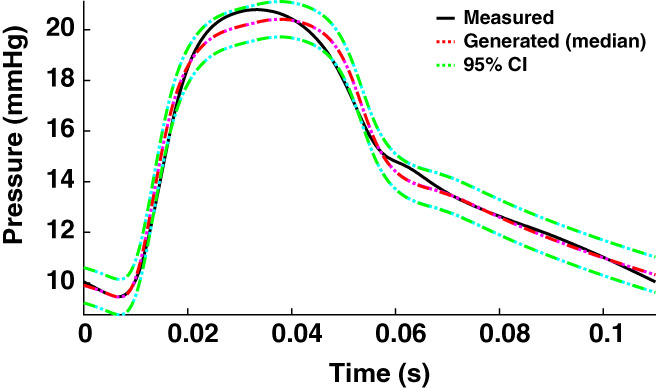
Superimposition of posterior median blood pressure time series obtained using all four emulation Hamiltonian and Lagrangian Dynamical Monte Carlo algorithms: GP AHMC, GP NUTS, GP ARMHMC, GP ALDMC (in red/magenta colours and different line types depending on the sampling algorithm used: red dotted/magenta dotted line for NUTS/HMC and red dashed/magenta dashed line for LDMC/RMHMC). With green/cyan colour and different line types depending on the sampling algorithm used (green dotted/cyan dotted line for NUTS/HMC and green dashed/cyan dashed line for LDMC/RMHMC) we show the 95% predictive credible interval, calculated analytically as *m*(***θ***) ± 1.96*σ*_total_, where *m*(***θ***) is the mean posterior pressure signal, and $\sigma_{\text{total}}=\sqrt{\sigma_{\text{error}}^{2}+\sigma_{\text{signal}}^{2}}$, with $\sigma_{\text{error}}^{2}$ being the mean posterior estimate for the noise variance, and $\sigma_{\text{signal}}^{2}$ being the variance of the distribution of the signals, which are model predictions obtained with the MCMC posterior parameter samples. Measured (physiological) blood pressure time series (black solid line) is superimposed. Note that the different medians and CIs corresponding to different algorithms are nearly indistinguishable, indicating great similarity in the results produced by the four methods

Next, we inspect the parameter estimation results. We have simulated the posterior median blood pressure time series (50% quantile) obtained from the simulations with the GP ALDMC sampler. We have superimposed the measured pressure waveform from the laboratory, obtained with right‐heart catheterisation. In addition, we have plotted the 95% predictive credible interval, calculated analytically as

$$m\left(\theta \right)\pm 1.96\sigma_{\text{total}},$$

where *m*(***θ***) is the mean posterior pressure signal, and

$$\sigma_{\text{total}}=\sqrt{\sigma_{\text{error}}^{2}+\sigma_{\text{signal}}^{2}},$$

with $\sigma_{\text{error}}^{2}$ being the mean posterior estimate for the noise variance, and $\sigma_{\text{signal}}^{2}$ being the variance of the distribution of the signals, which are model predictions obtained with the MCMC posterior parameter samples. The results are shown in the top panel of Figure 5. We notice that the 95% CI contains most of the measured data and that the measured and generated waveforms are in reasonable agreement, except for the peak, where a mismatch is registered. This points to the existence of a slight model mismatch, and in Section 7 we offer a potential explanation. In addition, in Figure A2 of the Appendix we include the posterior median pressure signal and the 95% CI for all methods: GP AHMC, GP NUTS, GP ARMHMC and GP ALDMC. We notice substantial overlap beyond the figure's resolution level between the medians and the CIs, indicating that all four algorithms provide very similar model predictions and UQ. This indicates agreement between the methods in output space.

#### 6.1.3 Posterior correlations

We have also investigated the existence of posterior correlations between the parameters (bottom panel of Figure 5) by plotting the pairwise scatterplots between the parameters. It appears that there is a moderate to strong positive correlation between the stiffness parameter, *s*, and the first resistance adjustment parameters, *r*_1_, as well as between the second resistance adjustment parameters, *r*_2_, and the compliance adjustment parameter, *c*.

#### 6.1.4 Efficiency

**FIGURE 6:**
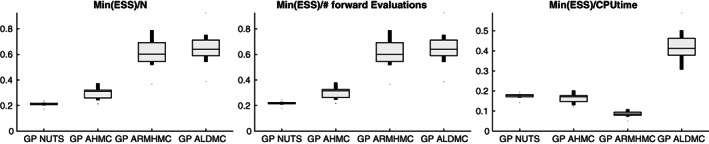
Measured (physiological) data inference results. Left panel: Distribution over 10 simulations of minimum Effective Sample Size (ESS) – Equation (20) – normalised by the total number of MCMC samples *N*. Middle panel: min(ESS) in Equation (20) normalised by the number of forward (PDE) evaluations. Right panel: min(ESS) in Equation (20) normalised by CPU time, for all algorithms tested, as part of the sampling phase in the GPHMC algorithm described in Section 4 for the measured (physiological) pulmonary blood pressure time series described in Section 2.3. Distribution obtained based on 10 chains, with 5000 sampling phase draws and 500 burn‐in draws, where the latter were discarded. Legend: NUTS: No U‐turn Sampler; AHMC: Adaptive Hamiltonian Monte Carlo; ARMHMC: Adaptive Riemann Manifold Hamiltonian Monte Carlo; ALDMC: Adaptive Lagrangian Dynamical Monte Carlo

For each of the emulation algorithms described in Section 3.2, we have run 10 simulations with different starting values for the parameters and different random number generator seeds. Table 3 summarises the simulation CPU time and the number of forward simulations (numerical PDE integrations) for every algorithm, the acceptance rate, the efficiency quantified using ESS, and the convergence in terms of MPSRF. Figure 6 also provides a comparison between these algorithms by displaying the distribution over 10 simulations of the minimum ESS normalised by the total number of MCMC samples *N*, and the minimum ESS is calculated by computing ESS for each of the four parameters, and taking the minimum from this set of 4:

(20) $${\mathrm{ESS}}^{*}=\min_{k}{\mathrm{ESS}}_{\mathrm{ik}},$$

where *i* = 1, …, 10 represents the simulation and *k* = 1, …, 4 the parameter label. We also show the minimum ESS in Equation (20) normalised by CPU time (i.e., the distribution based on 10 simulations of the minimum ESS normalised by the CPU time of each simulation), and the minimum ESS in Equation (20) normalised by the number of PDE evaluations for each of the 10 simulations. We note here that ESS^85^ estimates the effective number of independent samples out of the total number of MCMC samples, and a low value can indicate inefficiency of the sampler.

**CPU times**. All algorithms share the initial and the exploratory phase of the GPHMC algorithm. We thus start to compare the different algorithms in the sampling phase. The adaptive algorithms require pre‐processing, needed by the Bayesian optimisation scheme, while NUTS does not (Table 3). AHMC records somewhat higher CPU time than ALDMC in the pre‐processing and the sampling phase, despite the number of PDE evaluations required by each algorithm being somewhat similar (Table 3). This can be explained by the fact that the AHMC algorithm makes smaller steps and needs a larger number of steps for an efficient exploration of the parameter space when compared to ALDMC.‡‡ In addition, the CPU time is much larger for ARMHMC compared to ALDMC, despite the number of PDEs evaluated being the same. An explanation for this is the high computational costs of the numerical scheme utilised by ARMHMC to solve the Hamiltonian equations (see Section 3.2 for more details on this).

***ESS***. When looking at the minimum ESS, ESS^*^ (Equation (20)) normalised by the total number of MCMC samples *N* (left panel of Figure 6), we observe that the two algorithms which use gradient and curvature information from the log posterior distribution, namely ARMHMC and ALDMC, perform better than the algorithms which only use gradient information, namely NUTS and AHMC. It is clear that allowing the “mass matrix” **M** to adapt to the curvature of the log posterior distribution is highly beneficial. We note that the performance of ALDMC and ARMHMC is comparable, while AHMC registers a higher ESS than NUTS. A very similar trend is observed when analysing the minimum ESS in Equation (20) normalised by the number of PDE evaluations (middle panel of Figure 6). However, when the CPU time is taken into account (right panel of Figure 6), the ALDMC algorithm scores highest. In addition, ARMHMC pays the price of high computational costs, and is the least efficient algorithm in terms of CPU time. NUTS and AHMC have a similar performance, the advantage of NUTS is that no pre‐processing is required. In our case, the Bayesian optimisation scheme is not more efficient than the scheme employed by NUTS for the tuning of *L* and ɛ (when ESS normalised by CPU time is considered).

### 6.2 Synthetic data

We re‐run the analysis on synthetic data generated as described in Section 2.4 to investigate if the differences between methods hold for both synthetic and physiological data equally. The rationale for this comparison is to investigate how the model mismatch, which is only present in the real data but absent in the synthetic data, affects the inference and the performance of our methods.

#### 6.2.1 Accuracy

**FIGURE 7:**
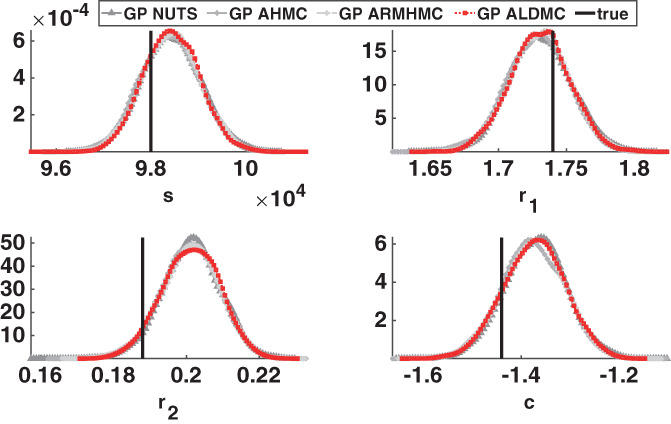
Synthetic data inference results. Kernel density estimation plots for the marginal posterior probability densities of the four parameters (*s*: stiffness, *r*_1_, *r*_2_, *c*: Windkessel parameters, see Equation (5)) for the GP emulation algorithms. To obtain the marginal posterior density, we have used the kernel smoothing function estimate for univariate data with the optimal bandwidth for normal densities.^90^ Samples were drawn using the MCMC algorithms from Section 3.2, which were combined with the GP emulator and classifier as described in Section 4. The data used are synthetic pulmonary pressure time series, generated as described in Section 2.4. The true parameter values which generated these data are superimposed as a black vertical line. Each simulation for the emulation algorithms consisted of 5000 sampling phase draws and 500 burn‐in draws (the latter were discarded). Legend: GP: Gaussian Process; NUTS: No U‐turn Sampler; AHMC: Adaptive Hamiltonian Monte Carlo; ARMHMC: Adaptive Riemann Manifold Hamiltonian Monte Carlo; ALDMC: Adaptive Lagrangian Dynamical Monte Carlo

Figure 7 shows the marginal posterior densities obtained with kernel density estimation from the posterior samples for all four emulation methods. We superimpose the true parameter values which generated the synthetic data in a black vertical line. The marginal posterior densities generated with the different methods overlap, and the true parameter values are contained within the marginal posterior densities for all four parameters, which is consistent with our inference procedure. In the Appendix A.6, we offer an explanation as to why the posterior densities are not centred at the true parameter values.

#### 6.2.2 Efficiency

**FIGURE 8:**
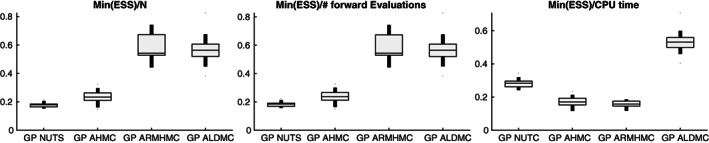
Synthetic data inference results. Left panel: Distribution over 10 simulations of minimum Effective Sample Size (ESS) – Equation (20) – normalised by the total number of MCMC samples *N*. Centre panel: min(ESS) in Equation (20) normalised by the number of forward (PDE) evaluations. Right panel: min(ESS) in Equation (20) normalised by CPU time, for all algorithms tested, as part of the sampling phase in the GPHMC algorithm described in Section 4 for the synthetic pulmonary blood pressure time series generated as described in Section 2.4. Distribution obtained based on 10 chains, with 5000 sampling phase draws and 500 burn‐in draws, where the latter were discarded. Legend: NUTS: No U‐turn Sampler; AHMC: Adaptive Hamiltonian Monte Carlo; ARMHMC: Adaptive Riemann Manifold Hamiltonian Monte Carlo; ALDMC: Adaptive Lagrangian Dynamical Monte Carlo

Figure 8 displays the distribution over 10 simulations of minimum ESS normalised by *N*, by the number of forward evaluations and by the CPU time. In terms of ESS normalised by *N* and number of model solutions, similar observations as for the physiological data can be made. NUTS remains the algorithm with the worst performance, AHMC is second worst and ARMHMC has a fairly similar performance to ALDMC. When analysing ESS/CPUtime, ALDMC remains the best algorithm, however, a larger discrepancy between AHMC and NUTS can be observed, with NUTS being clearly the second best. Interestingly, AHMC performs equally as poorly as ARMHMC.

## 7 DISCUSSION

### 7.1 Methodological approach

In the present study, we have combined GPs with state‐of‐the‐art MCMC schemes to enable parameter estimation and uncertainty quantification in an analytically non‐tractable, non‐linear system of coupled PDEs at manageable computational costs. This scheme differs from the approach of gradient matching, proposed for example, in Reference 91, where a GP is used to approximate the solutions of the differential equations and thereby avoid the computationally expensive numerical integration (see^92^ for the potential limitations). In our approach, there is no need to approximate the solution of the differential equations itself. Instead, we apply GP regression to approximate the likelihood function, with the objective to reduce the number of PDE evaluations required to calculate the likelihood during the MCMC iterations. We have evaluated various variants of this paradigm in terms of accuracy and efficiency on a biophysical fluid‐dynamics model of the pulmonary circulation. We have also built a GP classifier to automatically learn the regions in the parameter space that violate the physical assumptions of the mathematical model.

Our study presents several new methodologies. We have extended the GPHMC algorithm^32^ to allow for the use of other HMC variants (e.g., RMHMC), and we have coupled this with Bayesian optimisation for automatic parameter tuning. In addition, the combination of HMC and Delayed Acceptance^53^, ^55^ is novel. In terms of the application of our work, we have used fast Bayesian sampling methods to quantify uncertainty in the topical research area of quantitative physiology and pathophysiology, with a specific focus on the pulmonary circulation system, which is potentially interesting to clinical practitioners for detection of long‐term pulmonary hypertension. To our knowledge, employing an MCMC scheme and coupling it with GPs to emulate the likelihood, while performing approximate exact (meaning asymptotically converging to the true posterior probability distribution) inference has only been performed in one other study^61^ in the cardiovascular mathematical modelling field before, but with no extensive algorithm comparison. Most studies rely on parameter optimisation to estimate parameters,^3^, ^4^, ^5^, ^6^ which only provides a point estimate (i.e., a specific value) for the parameters, without allowing for an understanding of the uncertainty in these values. In addition, obtaining posterior distributions for the parameters allows an in‐depth understanding of the correlations between parameters, or construction and interpretation of credible intervals, which can reveal if the parameters can be used as disease indicators in the clinic.

### 7.2 Algorithm accuracy

Hamiltonian and Lagrangian algorithms (NUTS, AHMC, ARMHMC, ALDMC) have been employed, and they have all provided parameter posterior samples from approximately the same distribution, both for the physiological data study and synthetic data study (Figures 4 and 7 and Figure A1). The synthetic study results indicate that the true parameter values that generated the data are contained within the marginal posterior densities (Figure 7), which is consistent with our inference procedure. Additionally, the model predictions are very similar, all closely following the measured (physiological) data, and the UQ in output space is comparable (Figure A2) between algorithms. For the real data, for which the true parameter values are unknown, the agreement between the posterior probability densities in input space (Figure 4), and between model predictions and UQ in output space (Figure A2) across all algorithms is taken as a proxy for accuracy.

### 7.3 Posterior correlations

The existence of moderate to strong posterior correlations can be observed from Figure 5 (bottom panel). Analysing posterior correlations can reveal whether the model parameters can be uniquely identified from the data. Such insight into posterior parameter estimation uncertainty and correlation is important when considering whether the estimated parameter values are informative disease indicators to be used in a clinical decision support tool. However, such a tool needs to work in real time, and computational efficiency, which we discuss next, is therefore paramount.

### 7.4 Algorithm efficiency

#### 7.4.1 MPSRF versus ESS

The advantage of NUTS over the other algorithms is that it registers MPSRF < 1.1 while requiring zero pre‐processing effort. In addition, NUTS records the lowest CPU time in the sampling phase due to the lowest number of PDEs evaluated, while maintaining a large acceptance rate (97%). However, NUTS registers the poorest mixing and thus, lowest (worst) efficiency, as quantified by min(ESS)/N, which is the case for both the physiological and synthetic data studies. ESS is a measure of the algorithm's mixing, thus efficiency, by capturing the amount of information that the correlated MCMC samples contain about the target density, that is, ESS gives the equivalent number of independent samples out of the total number of MCMC correlated samples N. If the sampler exhibits poor mixing, it indicates that the Markov chain moves slowly through the parameter space, leading to low ESS relative to N. In that case, the samples drawn are highly correlated, and the sampler needs to be run for longer in order to acquire a sufficiently large number of independent samples and ensure a thorough exploration of the parameter space. In contrast, MPSRF indicates whether there are any signs of non‐convergence to the target density of the MCMC sampler. It is possible that the sampler appears to have converged to the target density, that is, MPSR<1.1, however the sampler's mixing, quantified via ESS, is poor, with high correlations between the MCMC samples, see our previous study^7^ for an example.

#### 7.4.2 ESS normalised by *N*

The algorithms which make use of curvature information from the log posterior distribution (ARMHMC, ALDMC) achieved higher efficiency (in terms of min(ESS)/*N*) than the algorithms which only use gradient information (AHMC, NUTS) for both the physiological and synthetic data studies. In applications from literature studies,^63^, ^64^, ^65^ and for the application presented here, (A)LDMC and (A)RMHMC use a larger step size and a smaller number of steps compared to (A)HMC and NUTS. The reason is that (A)LDMC and (A)RMHMC set the mass matrix based on the curvature of the log posterior, while (A)HMC and NUTS use an identity matrix as the mass matrix. The implication is that for (A)HMC and NUTS all the work falls on the back of the step size (and number of steps) to optimise efficiency, and the optimum step size is restricted by the lowest marginal variance. In contrast, for (A)LDMC and (A)RMHMC this issue is alleviated by the use of a non‐identity mass matrix. It is expected that the difference between (A)HMC/NUTS and (A)LDMC/(A)RMHMC is less pronounced for problems with similar marginal variances, for which the optimum step size would be similar across different parameters (see sec. 10.1.1 in Reference 63 for an example). The distance in space, ɛ × *L*, travelled by (A)LDMC and (A)RMHMC is generally larger than for (A)HMC and NUTS, leading to enhanced exploration efficiency of the former two compared to the latter two algorithms. This explains why min(ESS)/*N* is larger for ALDMC and ARMHMC compared to AHMC and NUTS. ALDMC and ARMHMC register fairly similar efficiency in terms of min(ESS)/*N*, and AHMC registers higher min(ESS)/*N* than NUTS for both data sets: physiological and synthetic. A potential explanation for the latter finding is that for NUTS the tuning of ɛ and *L* is performed in the emulated log posterior entirely, based on parameter samples accepted at the emulator stage, due to the construction of the algorithm, while for AHMC the tuning using Bayesian optimisation is based on samples accepted at the simulator stage, thus the simulator plays a role in finding the optimum tuning parameters, positively impacting efficiency. The finding that AHMC coupled with emulation has superior efficiency to NUTS with emulation is in line with the findings in Reference 65 comparing the conventional AHMC (i.e., without emulation) with the conventional NUTS.

#### 7.4.3 ESS normalised by CPU time

The efficiency and the acceptance rate of AHMC are enhanced at the cost of a larger number of PDEs being evaluated in the sampling phase, which results in larger CPU times compared to NUTS, thus in terms of min(ESS) normalised by CPU time, AHMC loses its advantage over NUTS for both the physiological and synthetic data studies. When comparing ALDMC to AHMC, the following can be noted. AHMC has the advantage of avoiding calculations of higher‐order derivatives, but requires a larger number of steps to be made, while ALDMC calculates higher‐order derivatives, but needs fewer steps. This appears to result into somewhat lower CPU times for ALDMC (Table 3), which coupled with a larger min(ESS), leads to ALDMC having a clear advantage in the case of physiological and synthetic data. Additionally, for both data sets, the ARMHMC algorithm loses its computational advantage due to the computationally burdensome integration scheme for the Hamiltonian dynamics (see Section 3.2), and scores the lowest. What is more, despite NUTS requiring a lower CPU time to run, ALDMC registers a min(ESS)/*N* nearly three times larger than NUTS, making the latter algorithm computationally most efficient in terms of ESS/CPU time.

#### 7.4.4 ESS normalised by number of forward evaluations

Regarding min(ESS) normalised by the number of forward evaluations, the same pattern can be observed as for min(ESS)/*N*, since the number of forward solutions is fairly similar to *N*.

Different dynamical systems are based on differential equations with different numerical integration costs. A computational complexity measure that is normalised by the number of forward simulations can be generalised across different dynamical systems, whereas other measures, like CPU time, cannot (as the differences in computational cost may merely reflect the differences in the numerical complexity of the underlying differential equations). Thus, the metric based on ESS/number of forward solutions can be used to compare strictly the performance of the algorithms, unlike ESS/CPU time. The comparative observations made about (A)HMC, NUTS, (A)LDMC and (A)RMHMC are based on empirical findings, to our knowledge no theoretical study comparing the efficiency between the algorithms has been performed.

#### 7.4.5 Real‐time treatment planning

Table 4 shows that the number of forward solutions per HMC iteration avoided by the coupling with a GP surrogate model is more than 100 on average. Therefore, a reduction in the number of forward solutions by two orders of magnitude is obtained. This demonstrates that using efficient surrogate models in place of the computationally expensive PDE model is key in assisting clinicians in real‐time treatment planning.

**TABLE 4:** Number of model evaluations (PDEs) required to obtain one single HMC sample drawn using conventional HMC versus emulation HMC (GPHMC) algorithm (mean and standard deviation in brackets)

Algorithm	Number of PDEs per HMC sample
Conventional HMC	117 (65)
Emulation HMC	1

*Note:* The number of PDEs for the conventional HMC is *L*(*d* + 1), where *L* is the number of leapfrog steps and *d* is the parameter dimensionality. The term *d* + 1 is the sum of one PDE evaluation to find the log likelihood, and *d* PDE evaluations to find the numerical derivatives by a first‐order differencing scheme with respect to each of the *d* parameters. For the fluid‐dynamics model, *d* = 4 and the mean and standard deviation of *L* are 23 (13). These statistics are based on an optimum *L* given by Bayesian optimisation. HMC is run with the number of leapfrog steps drawn from a uniform distribution with lower bound being 1 and upper bound being optimum *L*_._ ^65^ These results are based on measured (physiological) data.

#### 7.4.6 Summarising conclusions

In conclusion, given that ALDMC registers one of the two highest min(ESS) normalised by the total number of MCMC samples, one of the two highest min(ESS) normalised by the number of forward evaluations, and by far the highest min(ESS) normalised by CPU time, the empirical finding of our study is that in the context of the biophysical model investigated, the ALDMC algorithm with emulation is the best candidate for automatic clinical decision support. This conclusion holds for both physiological and synthetic data. In addition, considering the equivalent number of PDE evaluations replaced by the GP surrogate, it is clear that the emulation approach proposed is a key enabler for using the PDE model as a model‐based disease diagnostic in the clinic.

### 7.5 Limitations and future work

In the current study, the assumption of a constant stiffness parameter that is independent of the blood vessel dimension (Equation (2)), could be an oversimplification of the true physiological system. Thus, future work will include exploring a more complex physiological model which allows each pulmonary blood vessel to have its own wall stiffness parameter in a Bayesian hierarchical model, with a focus on formal model comparison to select the best model supported by the data. This modification will significantly increase the model complexity, possibly introducing multi‐modality in the posterior distribution.

HMC‐type algorithms work well for high‐dimensional posterior distributions, possibly with high correlations (see Chap. 5 in Reference 23 for a 100‐dimensional multivariate Gaussian distribution as a target density). For sampling from multi‐modal posteriors, HMC can be coupled with a tempering scheme (e.g., parallel tempering^93^), which entails sampling from a series of tempered distributions that are more diffuse than the target density (with flattened out modes), enabling the sampler to traverse the regions of low probability between the modes.^94^

The increase in parameter complexity could lead to a much larger number of training points required to train the GP emulator to ensure a dense enough coverage of the parameter space. O'Hagan^12^ notes that GP emulation is likely to be implemented effectively with up to 50 inputs on modern computing platforms. The number of training points required to optimally cover a 50D input space depends on the complexity of the problem (e.g., smoothness of the log posterior), and is restricted by the O(*n*^3^) computational complexity of GP emulators (due to the covariance matrix inversion). Conventional GPs may be replaced by sparse GPs,^95^ which optimally select a lower number of training points while retaining the maximum information at reduced computational costs.

In addition, in future studies we will use GPs to model the correlation of the measurement errors,^6^ caused by the temporal nature of the data, and by the smoothing and averaging. We will also model the apparent model mismatch, related to the discrepancy between the real‐world system and the simulator output (see the peak shift in the top panel of Figure 5). The model discrepancy may be caused by errors from the numerical integration of the PDEs, the assumption of purely elastic vessel walls, pressure and flow waveforms that do not have a direct physical relationship (due to the data smoothing and averaging), or by the strict periodicity assumption of the measured pressure and flow (Equation (3)).

Ignoring model discrepancy can potentially lead to an underestimation of the uncertainty and a bias in the predictions – see^11^, ^14^, ^96^, ^97^, ^98^ for further details. With the focus on computational efficiency and comparative efficiency evaluation, studying this in more detail is beyond the remit of the present work. However, in future work, we will systematically identify all sources of potential model limitations, including assumptions made about the pulmonary arterial network geometric parameters (e.g., vessels' radii, vessels' lengths), the vessels' connectivity (i.e., vessel bifurcations, trifurcations), and the network size (i.e., number of vessels).^99^ We will then follow^14^, ^96^ and apply a separate GP to model and quantify this discrepancy. This will lead to a certain increase in the computational complexity, and will thus benefit substantially from the efficiency evaluation and improvement carried out in the present work.

### 7.6 Final remarks

In conclusion, biophysical parameters of mathematical cardiovascular models have genuine predictive value for disease prognostication, as demonstrated in Reference 100. However, fast parameter estimation and computational efficiency are paramount for clinical decision making. Our benchmark study comparing several state‐of‐the‐art sampling methods, particularly adapted to complex and expensive computational models sheds light on their relative computational efficiencies. This is a stepping stone towards a decision support system for personalised medicine that can help clinical practitioners to make informed decisions in real time.

Parts of the work reported in this paper were presented by the first author at the annual STEM for BRITAIN competition at the Houses of Parliament in Westminster, UK on March 9, 2020, where they won the gold medal in the category “Mathematics”; see http://www.setforbritain.org.uk/2020event.asp for details.

## Figures and Tables

**FIGURE A3:**
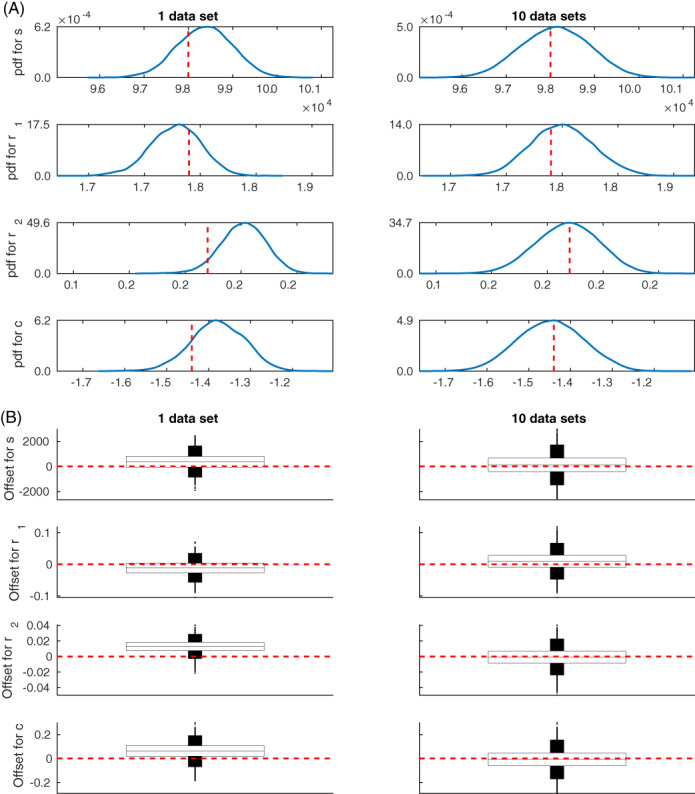
Synthetic data inference results. Panel (A): Kernel density estimation plots for the marginal posterior probability densities of the four parameters (*s*: stiffness, *r*_1_, *r*_2_, *c*: Windkessel parameters). Five thousand posterior samples are drawn using the GP emulation Hamiltonian Monte Carlo algorithm for every synthetic data set. The left side plots are based on the posterior samples from 1 data set, and the right side plots are based on agglomerated posterior samples from 10 data sets. The true parameter values are superimposed as vertical red lines. Panel (B): Boxplot representation of the distribution of offsets for the parameters, calculated using Equation (A13) for 1 and 10 data sets. The horizontal red line marks zero offset

## Endnotes

*
First‐order finite difference scheme: $\frac{\mathrm{df}\left(x\right)}{\mathrm{dx}}=\frac{f\left(x+h\right)-f\left(x\right)}{h}$, where *h* > 0 is a small constant.

†
The PDE system is augmented to include the gradient of the states with respect to the parameters (called the sensitivity derivative equation).

‡
Avoids solving the sensitivity derivative equation by using a linear adjoint equation, which is easier to numerically integrate.

§
The likelihood is tractable, thus Bayesian methods such as Approximate Bayesian Computation (ABC)^59^ or pseudo‐marginal MCMC^60^ are not required here.

¶
In the rare case that a move into the invalid regime gets accepted, a value based on GP extrapolation will be used for the surrogate log posterior. This value is unlikely to be in good agreement with the true log posterior. However, recall from the previous discussion that such a mismatch only affects the computational efficiency of the sampling algorithm, not its accuracy. A poor match between the true and emulated posteriors leads to a low acceptance probability in Equation (14), and thus low efficiency.

**
For that reason, we replace the squared exponential kernel, which is infinitely differentiable, by the Matérn class kernel, which gives a prior distribution that assumes the functions are only once differentiable.

††
We checked the analytical derivative against the numerical derivative; a difference in Euclidean space below a small threshold, for example, 0.1%, is taken as an indication that the two are in agreement.

‡‡
The Bayesian optimisation converges to ɛ = 0.005 ± 0.0004, *L* = 48 ± 3 for AHMC, and ɛ = 0.078 ± 0.004, *L* = 38 ± 4 for ALDMC.

## Brief review of surrogate modelling

Surrogate models can be split into three categories:

- Data‐driven models (data fit interpolation and regression models) are constructed by an input–output map for the original (high‐fidelity) model, and methods to do this include polynomial chaos expansion,^41^ Gaussian Processes (GPs), also known as Kriging,^35^, ^42^, ^72^, ^73^, ^74^, ^101^ multivariate adaptive regression splines,^102^ random basis functions,^33^neural networks,^103^ support vector machines^104^; for a review on data‐driven surrogate models, the reader is referred to.^105^, ^106^, ^107^
- Projection‐based reduced models reduce the parameter space dimensionality by projecting the governing equations into a basis of orthonormal vectors – see^106^ for a review and^108^, ^109^ for an application to structural dynamics, and to large‐scale statistical inverse problems.
- Multi‐fidelity (hierarchical) models are surrogate models created from the original (high‐fidelity, complex) model by reducing the numerical resolution, or by simplifying the physical process – see^106^ for a review and^40^, ^110^ for an application to cardiac electrophysiology.

Given that the fluid‐dynamics model under consideration has a fairly low number of parameters (4), in our work we use GPs, a probabilistic inference method reviewed in Appendix A.2, to construct a surrogate for the data log likelihood. We note that the data likelihood points used to construct the surrogate model are calculated based on the original, high‐fidelity fluid‐dynamics model (i.e., the original governing fluid‐dynamics equations are used).

## Gaussian processes

This section gives a brief overview of GPs. For a detailed introduction to GPs, the reader is referred to.^74^

### Gaussian processes for regression

A stochastic process $f=f{\left(x\right)}_{x\in X}$ is defined as a Gaussian process if the random variables **f** = (*f*(**x**_1_), …, *f*(**x**_*n*_)) are jointly normal for any inputs **x**_*i*_ ∈ ℝ^*d* × 1^, with *i* = 1, …, *n*, $f∼ℳ\mathrm{VN}\left(m,K\right),$ where **m** = (*m*(**x**_1_), …, *m*(**x**_*n*_)) is the mean *n*‐vector and $K={\left[k\left(x_{i},x_{j}\right)\right]}_{i,j=1}^{n}$ is the *n* × *n* variance–covariance matrix of **f**. In GP models,^74^, ^111^ inputs **X** = [**x**_1_, …, **x**_*n*_]^*T*^ (**X** is an *n* × *d* matrix) are mapped into outputs **y** = (*y*_1_, …, *y*_*n*_) (**y** is an *n*‐vector) by means of latent noiseless functions **f**. A GP prior is placed on the distribution of these functions as a way to account for the uncertainty in the functional form. Assuming iid Gaussian noisy observations **y**:

(A1) $$\begin{array}{l} y|f∼ℳ\mathrm{VN}\left(f,\sigma^{2}I\right),\\ f\left(X\right)|\gamma ∼\mathrm{GP}\left(m\left(X\right),K|\gamma \right),\\ \gamma ,\sigma^{2}∼p\left(\gamma \right)p\left(\sigma^{2}\right), \end{array}$$

where **γ** contains the covariance function (kernel) hyperparameters, and *σ*^2^ is the observation noise variance. Typically, the mean of the GP is set to zero (**m**(**X**) = **0**), that is, the data are standardised to zero mean, which will henceforth be assumed. The covariance function, *k*(**x**, **x** '  ∣ **γ** ), gives the smoothness and variability of the latent functions. Several covariance functions are available; see for example, Chapter 4 in Reference 74 for a review. The kernel hyperparameters **γ** and the observation noise variance *σ*^2^ can be placed a prior on and integrated out from the joint posterior distribution *p*(**f**, **γ**, *σ*^2^| *D*), where *D* = {**X**, **y**} is the set of training points, or they can be found in a so‐called empirical Bayes approach by maximisation of the log marginal likelihood,

(A2) $$\log p\left(y|X,\gamma ,\sigma^{2}\right)=-\frac{n}{2}\log\left(2\pi \right)-\frac{1}{2}\log|K+\sigma^{2}I|-\frac{1}{2}y^{T}{\left(K+\sigma^{2}I\right)}^{-1}y.$$

The aim of constructing a probabilistic model using GPs is prediction at unseen input values. The predictive distribution of a new function, $\tilde{f}=f\left(\tilde{x}\right)$, is also Gaussian,^74^ as follows:

(A3) $$\tilde{f}|D,\gamma ,\sigma^{2}∼N\left(m_{p}\left(\tilde{x}\right),k_{p}\left(\tilde{x},{\tilde{x}}^{'}|\gamma \right)\right),$$

(A4) $$m_{p}\left(\tilde{x}\right)=k\left(\tilde{x},X|\gamma \right){\left(K+\sigma^{2}I\right)}^{-1}y,$$

(A5) $$k_{p}\left(\tilde{x},{\tilde{x}}^{'}|\gamma \right)=k\left(\tilde{x},{\tilde{x}}^{'}|\gamma \right)-k\left(\tilde{x},X|\gamma \right){\left(K+\sigma^{2}I\right)}^{-1}k\left(X,{\tilde{x}}^{'}|\gamma \right),$$

where $k\left(\tilde{x},X|\gamma \right)$ is a vector valued kernel function, $k\left(\tilde{x},X\right):{\mathbb{R}}^{d\times 1}\times {\mathbb{R}}^{d\times n}\to {\mathbb{R}}_{+}^{1\times n}$, $k\left(X,\tilde{x}|\gamma \right)$ is a vector valued kernel function, $k\left(X,\tilde{x}\right):{\mathbb{R}}^{d\times n}\times {\mathbb{R}}^{d\times 1}\to {\mathbb{R}}_{+}^{n\times 1}$, and the hyperparameters **γ** and *σ*^2^ have been obtained based on the training data (**X**, **y**).

The derivative of the posterior predictive mean and variance with respect to the new input point $\tilde{x}=\left({\tilde{x}}_{1},\ldots ,{\tilde{x}}_{d}\right)$ can be computed analytically, and for example, the first order partial derivatives are:

(A6) $$\frac{\partial m_{p}\left(\tilde{x}\right)}{\partial {\tilde{x}}_{j}}=\frac{\partial k\left(\tilde{x},X|\gamma \right)}{\partial {\tilde{x}}_{j}}{\left(K+\sigma^{2}I\right)}^{-1}y,$$

(A7) $$\frac{\partial k_{p}\left(\tilde{x},\tilde{x}|\gamma \right)}{\partial {\tilde{x}}_{j}}=\frac{\partial k\left(\tilde{x},\tilde{x}|\gamma \right)}{\partial {\tilde{x}}_{j}}-\frac{\partial k\left(\tilde{x},X|\gamma \right)}{\partial {\tilde{x}}_{j}}{\left(K+\sigma^{2}I\right)}^{-1}k\left(X,\tilde{x}|\gamma \right)-k\left(\tilde{x},X|\gamma \right){\left(K+\sigma^{2}I\right)}^{-1}\frac{\partial k\left(X,\tilde{x}|\gamma \right)}{\partial {\tilde{x}}_{j}},$$

where

(A8) $${\left(\frac{\partial k\left(\tilde{x},X|\gamma \right)}{\partial {\tilde{x}}_{j}}\right)}^{T}=\left[\begin{array}{c} \frac{\partial k\left(\tilde{x},x_{1}|\gamma \right)}{\partial {\tilde{x}}_{j}}\\ \vdots \\ \frac{\partial k\left(\tilde{x},x_{n}|\gamma \right)}{\partial {\tilde{x}}_{j}} \end{array}\right]\;\text{and}\;\frac{\partial k\left(X,\tilde{x}|\gamma \right)}{\partial {\tilde{x}}_{j}}=\left[\begin{array}{c} \frac{\partial k\left(x_{1},\tilde{x}|\gamma \right)}{\partial {\tilde{x}}_{j}}\\ \vdots \\ \frac{\partial k\left(x_{n},\tilde{x}|\gamma \right)}{\partial {\tilde{x}}_{j}} \end{array}\right].$$

Derivatives may be needed if GPs are coupled with methods which require knowledge of the derivatives, for example, the GPHMC algorithm.^32^

In the present paper, we follow the work of^11^, ^72^, ^73^ and use GPs for statistical emulation of the unnormalised log posterior distribution, as described in more detail in Section 4.

### Gaussian processes for classification

Besides recording a real‐valued dependent variable, we may record a binary variable ***λ*** = (*λ*_1_, …, *λ*_*n*_) with *λ*_*i*_ ∈ {0, 1} associated with inputs $X={\left\{x_{i}\right\}}_{i=1}^{n}$, where 0: failure and 1: success. GPs can also be applied to classification problems, where we are for example interested in deciding on the success of the PDE evaluation for a particular parameter configuration. To do so, we can build a GP classification model that places a GP prior over the distribution of noiseless latent functions, **f**, as follows:

(A9) $$\begin{array}{l} \lambda_{i}|f\left(x_{i}\right)∼\text{Bernoulli}\left(p\left(\lambda_{i}=1|f\left(x_{i}\right)\right)\right),\;\\ f\left(X\right)|\gamma ∼\mathrm{GP}\left(m\left(X\right),K|\gamma \right),\;\\ \gamma ∼p\left(\gamma \right),\; \end{array}$$

where **γ** contains the kernel hyperparameters.

The binary observations (class labels), ***λ***, are drawn from a Bernoulli distribution with a success probability *p*(*λ*_*i*_ = 1| *f*(**x**_*i*_)), and likelihood shown in Equation (A11). The success probability is related to the function *f*(**x**_*i*_) via the sigmoid function, *sig*(*f*(**x**_*i*_)) = (1 + exp(−*f*(**x**_*i*_)))^−1^, which transforms the probability into the unit interval, [0,1], as shown in Equation (A10):

(A10) $$p\left(\lambda_{i}=1|f\left(x_{i}\right)\right)=\mathrm{sig}\left(f\left(x_{i}\right)\right);\;p\left(\lambda_{i}=0|f\left(x_{i}\right)\right)=1-p\left(\lambda_{i}=1|f\left(x_{i}\right)\right),$$

(A11) $$\text{Likelihood}:p\left(\lambda_{i}|f\left(x_{i}\right)\right)={\left(\mathrm{sig}\left(f\left(x_{i}\right)\right)\right)}^{\lambda_{i}}{\left(1-\mathrm{sig}\left(f\left(x_{i}\right)\right)\right)}^{\left(1-\lambda_{i}\right)}\;\text{for}\;a\;\text{binary outcome}\;\lambda_{i}\in \left\{0,1\right\}.$$

The latent functions, **f**, can be integrated out from the conditional posterior distribution given the hyperparameters **γ**, to obtain the marginal likelihood (Equation (A12)), which has no closed‐form solution:

(A12) $$p\left(\lambda |X,\gamma \right)=\int p\left(\lambda ,f|X,\gamma \right)df=\int p\left(\lambda |f\right)p\left(f|X,\gamma \right)df.$$

With a non‐Gaussian likelihood, we can run MCMC,^112^ or approximate the conditional posterior distribution by a Gaussian form using the Laplace approximation,^113^ variational inference^114^ or expectation propagation (EP).^115^

## Log posterior differentiability for HMC

In HMC‐type algorithms, the log posterior distribution must be differentiable with respect to the parameters. For example, in some cardiovascular models with parameters related to the heart valves and the timing wave propagation phenomena, the log posterior, while being a continuous function, may not be differentiable with respect to the parameters. This issue can be overcome by using an HMC method with a modified leapfrog scheme, in which the gradient of the log posterior is replaced by the proximity operator.^116^ Non‐differentiability is only an issue if HMC without emulation is employed, whereas when HMC is coupled with emulation, smoothness, thus differentiability, is controlled by the choice of the GP kernel.

For GPHMC, calculating the log posterior derivatives requires finding derivatives of the GP predictive mean and variance, and these expressions are available in analytical form (Equations (A6) and (A7)). In contrast, HMC requires derivatives for the simulator, which are in most cases obtained numerically (provided differentiability holds), and that requires several considerations to be made (e.g., differencing scheme used: first/second order, choice of discretisation step). These choices may affect the performance of the HMC algorithm, for example for rough signals, the use of first‐order differencing (which is less accurate than central differences, i.e., second‐order) could lead to a sub‐optimal HMC sampler, with reduced exploration efficiency, since unreliable derivative estimates introduce noise into the Hamiltonian equations. For further details, for example, on selecting the discretisation step in the finite difference scheme, see Chap. 4 and fig. 4.5 in Reference 117. These issues only need considering when HMC without emulation is used, however they are avoided in our work, where HMC is coupled with emulation using GPs, since the derivatives are available in analytical form.

## Parameter pairwise scatterplots

Figure A1 shows the marginal posterior densities and the pairwise scatterplots, obtained based on the posterior samples drawn using all emulation Gaussian Process Hamiltonian and Lagrangian Dynamical Monte Carlo algorithms for the measured data study.

## Model predictions and UQ

Figure A2 displays the model predictions (posterior median signals), as well as the 95% credible intervals obtained with each of the emulation Gaussian Process Hamiltonian and Lagrangian Dynamical Monte Carlo algorithms.

## Parameter inference and UQ for multiple synthetic data sets

Figure 7 presents results based on one synthetic data set. To generate the data set, one noise instantiation was added to the prediction from the PDE model. With one noise realisation, we may obtain an offset from the true parameter values. This offset is expected to be increasingly reduced when computed based on agglomerated posterior samples from multiple data sets generated with different noise instantiations. Thus, in expectation (over different data sets), the true parameter values are recovered

To demonstrate this, we have performed the inference analysis by running the emulation HMC algorithm on 10 different data sets generated with different noise instantiations. We combine the posterior samples from all data sets, and the aim is to illustrate that as a larger number of data sets is used, the parameters are more accurately estimated. In Figure A3(A) we display the marginal posterior probability density of the parameters obtained with kernel density estimation. The left side plots are based on posterior samples from 1 data set, and the right side plots are based on agglomerated posterior samples from 10 data sets. Additionally, we calculate the offset for the 4‐dimensional parameter vector ***θ*** for 1 and 10 data sets, by subtracting the true parameter from the agglomerated posterior samples (i.e., posterior samples combined from all *K* data sets) as follows:

(A13) $$d\left(\theta \right)=\theta_{\cup_{k=1}^{K}}-\theta_{\text{true}},\;K=\left\{1,10\right\},$$

where $\theta_{\cup_{k=1}^{K}}$ is the union of *K* sets of parameter posterior samples corresponding to the *K* data sets. In Figure A3(B) we show the distribution of offsets.

A clear offset is registered when one single data set is used, and this offset gets reduced when the agglomerated posterior samples from 10 data sets are shown. It is straightforward to prove for analytically tractable toy problems (e.g., a binomial distribution used to describe tossing a coin) that the offset converges to zero as the number of independent data instantiations goes to infinity. In principle this could be shown numerically for our problem by increasing the number of independent data instantiations, but the computational costs would be excessive.
